# A mosquito lipoxin/lipocalin complex mediates innate immune priming in *Anopheles gambiae*

**DOI:** 10.1038/ncomms8403

**Published:** 2015-06-23

**Authors:** Jose Luis Ramirez, Giselle de Almeida Oliveira, Eric Calvo, Jesmond Dalli, Romain A. Colas, Charles N. Serhan, Jose M. Ribeiro, Carolina Barillas-Mury

**Affiliations:** 1 Laboratory of Malaria and Vector Research, National Institute of Allergy and Infectious Diseases, National Institutes of Health, Rockville, Maryland 20852, USA; 2 Center for Experimental Therapeutics and Reperfusion Injury, Department of Anesthesiology, Perioperative, and Pain Medicine, Brigham and Women’s Hospital and Harvard Medical School, Boston, Massachusetts 02115, USA

## Abstract

Exposure of *Anopheles gambiae* mosquitoes to *Plasmodium* infection enhances the ability of their immune system to respond to subsequent infections. However, the molecular mechanism that allows the insect innate immune system to ‘remember’ a previous encounter with a pathogen has not been established. Challenged mosquitoes constitutively release a soluble haemocyte differentiation factor into their haemolymph that, when transferred into Naive mosquitoes, also induces priming. Here we show that this factor consists of a Lipoxin/Lipocalin complex. We demonstrate that innate immune priming in mosquitoes involves a persistent increase in expression of Evokin (a lipid carrier of the lipocalin family), and in their ability to convert arachidonic acid to lipoxins, predominantly Lipoxin A_4_. *Plasmodium* ookinete midgut invasion triggers immune priming by inducing the release of a mosquito lipoxin/lipocalin complex.

## Abstract (web summary)

A soluble factor induced by *Plasmodium* infection promotes hemocyte differentiation and increases mosquitoe resistance to subsequent infections. Here the authors show that this factor consists of a Lipocalin/Lipoxin A4 complex, and that insects can metabolize arachidonic acid to produce lipoxins.

## Graphical abstract

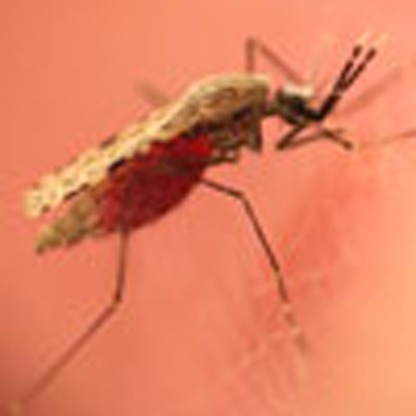

Memory-like responses have been described in several insect species, a phenomenon that has been termed ‘immune priming’^1^,^2^,^3^,^4^,^5^. For example, pre-exposure of *Anopheles gambiae* mosquitoes to *Plasmodium* infection, in the presence of the gut microbiota, enhances the immune response to subsequent infections^2^. In both *Drosophila*^1^ and mosquitoes^2^, haemocytes have been shown to be essential for enhanced immunity after a challenge. Natural killer cells are key mediators of vertebrate innate immune responses and have also been shown to be capable of immunological memory^6^. Immunological memory in the adaptive immune system involves genomic rearrangements and/or clonal expansion of long-lived memory cells. However, the molecular mechanism of immune priming in insects has not been established. *Plasmodium*-challenged mosquitoes constitutively release a soluble haemocyte differentiation factor (HDF) into their haemolymph^2^,^7^ that induces priming when transferred into Naive mosquitoes^2^. Here we describe the identification of HDF as a Lipoxin/Lipocalin complex, and show that innate immune priming involves a persistent increase in the ability of mosquitoes to convert arachidonic acid to lipoxins, as well as higher expression of their lipid carrier, a member of the lipocalin family that we named Evokin.

## Results

### Biochemical properties of HDF

**Figure 1:**
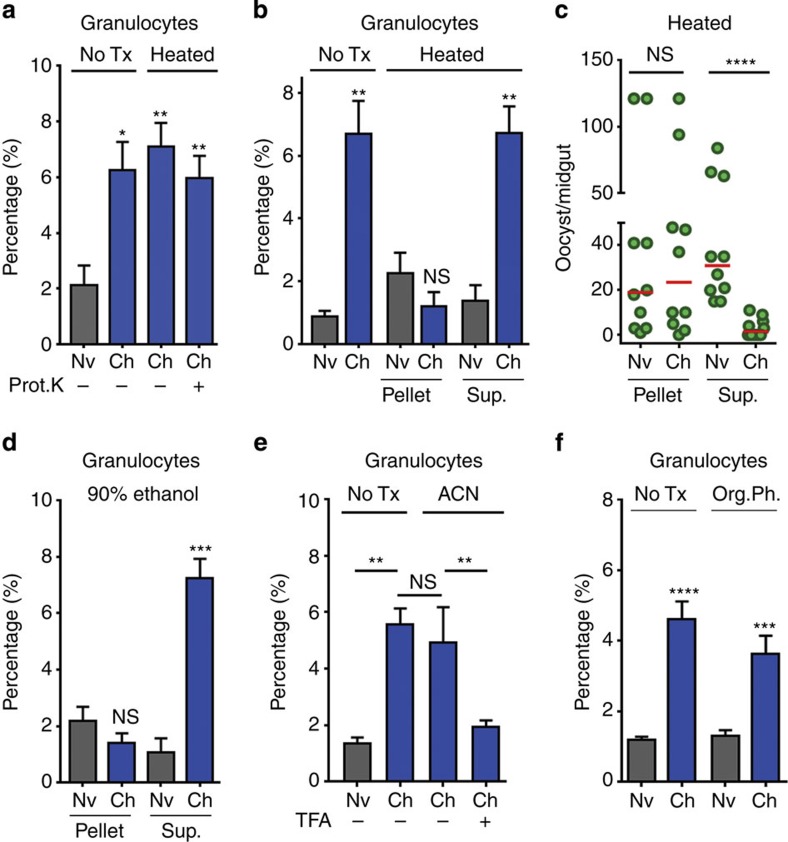
Biochemical properties of HDF. (**a**) Effect of proteinase K and heat treatment on HDF activity, indicated by an increase in the proportion of granulocytes in recipient mosquitoes. (**b**) Bioactivity of pellet and supernatant fractions from heat-treated haemolymph evaluated 4 days post injection. (**c**) Susceptibility to *P. berghei* infection 4 days after systemic injection of pellet and supernatant fractions from heat-treated haemolymph. HDF activity following (**d**) ethanol precipitation, (**e**) TFA treatment and (**f**) lipid extraction. ACN, Acetonitrile; Ch, Challenged; Nv, Naive; Sup, Supernatant; Tx, Treatment. Error bars in **a**,**b** and **d**–**f** represent mean±s.e.m. Granulocyte proportions were determined for each individual mosquito. Haemocytes were counted in 6–10 mosquitoes for each treatment and the results were confirmed in at least 2 independent experiments. Each circle in **c** represents the number of parasites in an individual midgut and the line indicates the medians. Mann–Whitney test, *P≤0.05; **P≤0.01; ***P≤0.001; ****P≤0.0001; NS, *P*>0.05.

The stability and biochemical nature of HDF was explored using a bioassay in which haemolymph from Challenged mosquitoes was collected 5–7 days post infection and subjected to different treatments before it was injected into Naive recipients. The ability of HDF to induce an increase in granulocytes was evaluated after each treatment. Mosquito HDF is very stable and remained active when cell-free haemolymph was heated (70 °C for 30 min) or was treated with proteinase K, followed by heat inactivation (80 °C for 30 min) (Fig. 1a; Supplementary Fig. 1a,b). Heat-treated HDF also enhanced antiplasmodial immunity (Fig. 1b,c); and HDF remained in solution and active when the heat treatment was followed by ethanol precipitation (Fig. 1d; Supplementary Fig. 1c). It was also soluble and active after exposure to acetonitrile, but the activity was completely lost if acetonitrile was acidified with trifluoroacetic acid (Fig. 1e). When the haemolymph of Challenged mosquitoes was subjected to extraction with organic solvents (methanol/chloroform), biological activity could be recovered in the organic phase (Fig. 1f), indicating that HDF might be a lipid.

HDF in aqueous solution was loaded into a C18 TARGA Minispin column and its chromatographic properties were evaluated using step elutions with increasing concentrations of acetonitrile. The interaction of HDF with the C18 matrix was weak and the biological activity eluted with the first elution steps (Supplementary Fig. 2). When a similar strategy was used to evaluate the interaction with a hydrophilic chromatography matrix, using a normal phase Hydrophilic Interaction Liquid Chromatography (HILIC) tip column at pH 6.5, the interaction was stronger (Supplementary Fig. 3). We reasoned that HDF could have more than one component, for example, it might consist of a protein–lipid complex, with a protein carrier that would provide a hydrophilic surface and explain the observed chromatographic behaviour.

### Identification of the protein component of HDF

**Figure 2:**
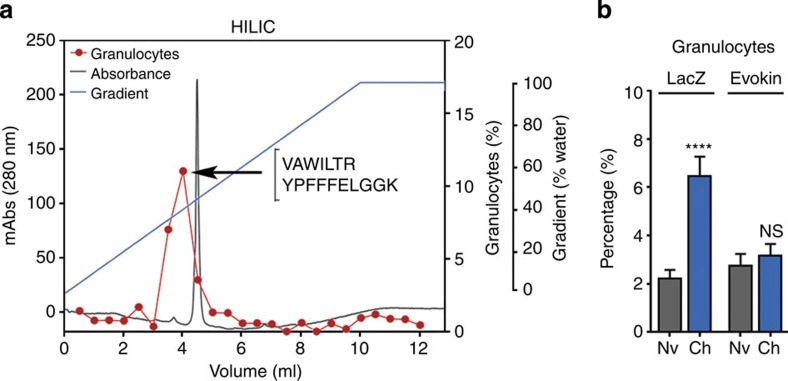
Identification of the protein component of HDF. (**a**) Haemolymph Hydrophilic Interaction Liquid Chromatography (HILIC), protein absorbance (black line), HDF activity in HILIC fractions (% granulocytes after injection of recipient mosquitoes) (red line) and acetonitrile to water gradient (blue line). Peptide sequences in the fraction with the highest biological activity indicated by the arrow. (**b**) Effect of silencing Evokin on haemolymph HDF activity, indicated by an increase in the proportion of granulocytes 4 days after systemic injection of haemolymph from Naive (Nv) and Challenged (Ch) mosquitoes in which Evokin was present or absent. Error bars represent the mean±s.e.m. Granulocyte proportions were determined for each individual mosquito. Haemocytes were counted in 6–10 mosquitoes for each treatment and the results were confirmed in at least 2 independent experiments. Mann–Whitney test, ****P≤0.0001; NS, *P*>0.05.

To identify the putative protein component, haemolymph was collected from *Plasmodium berghei*-challenged mosquitoes and the soluble fraction after acetonitrile precipitation was subjected to HILIC. Fractions were collected and assayed for biological activity (Fig. 2a). The fraction with the highest activity was subjected to matrix-assisted laser desorption/ionization (MALDI)-time-of-flight (TOF)-mass spectrometry (MS) analysis. Two peptide sequences were present in this fraction (VAWILTR and YPFFFELGGK), and both had a perfect match to an *A. gambiae* protein with homology to vertebrate lipocalins. We named this protein ‘Evokin’ (AGAP009281-PA) (Supplementary Table 1). Evokin expression in response to *Plasmodium* infection increased significantly (4-fold) in the abdominal wall 24 h post feeding (PF) (*P*<0.01) and remained elevated (1.75-fold) at 7 days PF (*P*<0.001) (Supplementary Fig. 4). In contrast, midgut expression in response to infection at 24 h and 7 days PF was not significantly different from uninfected controls (Supplementary Fig. 4). Furthermore, transfer of haemolymph from Evokin-silenced Challenged mosquitoes no longer induced haemocyte differentiation, nor did it enhance immunity in the recipients (Fig. 2b), confirming that Evokin is a critical component of HDF.

The lipocalins are a family of proteins that transport small hydrophobic molecules such as steroids, retinoids and lipids^8^,^9^,^10^. They are present in many diverse species including bacteria, insects and vertebrates, and are characterized by a low sequence similarity but a highly conserved protein structure (reviewed in the study by Flower^10^). Fruit fly lipocalins participate in JNK-dependent metabolic stress responses and in development of the nervous system^8^,^10^,^11^. Lipocalins have also been proposed to protect their ligands from oxidative degradation, to provide target specificity to their cargo, and to regulate ligand release from the sites of synthesis^8^,^10^

### Identification of the lipid component of HDF

**Figure 3:**
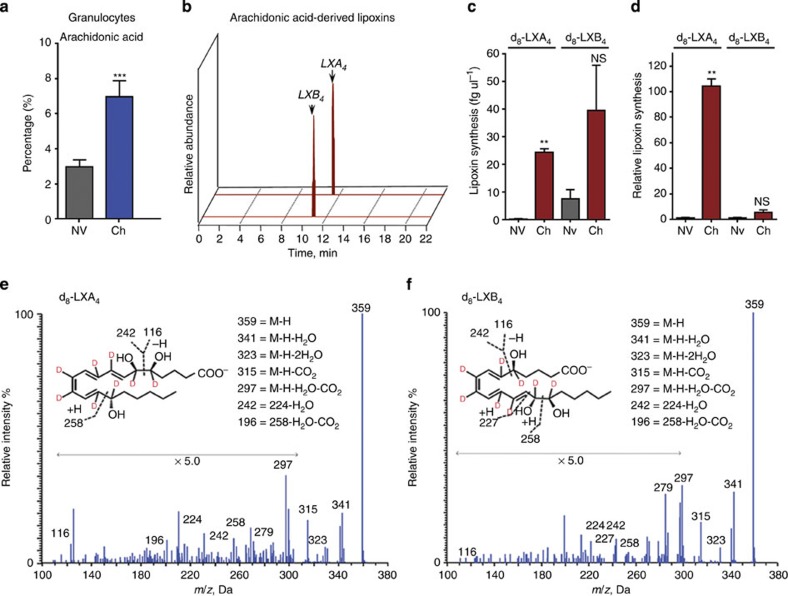
Synthesis of Lipoxins (LX) in mosquitoes following arachidonic acid administration. (**a**) Effects of arachidonic acid injection on HDF activity (% granulocytes) in haemolymph from Naive (Nv) and Challenged (Ch) mosquitoes. Haemocytes were counted in 6–10 mosquitoes for each treatment and the results were confirmed in at least 2 independent experiments. Bars represent the mean±s.e.m. Mann–Whitney test, ***P≤0.001. (**b**) Representative chromatographs obtained by Multiple Reaction Monitoring (MRM) of the parent ion (Q1) *m/z* 359 and a diagnostic daughter ion (Q3) for deuterium-labelled (d_8_) LXA_4_ and LXB_4_ (**c**) d_8_-LXA_4_ and d_8_-LXB_4_ levels in Naive and Challenged mosquitoes. (**d**) d_8_-LXA_4_ and d_8_-LXB_4_ lipoxin synthesis in Challenged mosquitoes relative to Naive controls. Bars for **c**,**d** represent mean±s.e.m. Student’s *t-*test, **P≤0.01; NS, *P*>0.05. MS/MS fragmentation spectrum employed for the identification of (**e**) d_8_-LXA_4_ and (**f**) d_8_-LXB_4_. Results for **b**–**f** are representative of two independent haemolymph preparations.

To search for the potential lipid ligand of Evokin, haemolymph from Challenged mosquitoes was subjected to the same HILIC chromatography procedure, and equivalent fractions were assayed for biological activity (Supplementary Fig. 5). Lipids were extracted from the fraction with the highest activity (B4; Supplementary Fig. 6a) and the preceding fraction (B3) lacking activity, and both subjected to lipid mediator (LM) lipidomics (Supplementary Fig. 6). Two peaks were prominent in the active fraction (Supplementary Fig. 6b) that were absent in the inactive one (Supplementary Fig. 6c). MS/MS spectrum analysis (Supplementary Fig. 7) of these peaks identified a lipid that shares some mass spectrum signatures with vertebrate lipoxins (Supplementary Fig. 7a); and a second one corresponded to 4-hydroxy-docosahexaenoic acid (4-HDHA) (Supplementary Fig. 7b). However, the limited amount of material precluded a definitive identification. Because lipoxins are derived from arachidonic acid, we decided to perform a comparative LM metabololipidomic analysis between haemolymph of Naive and Challenged mosquitoes, after systemic administration of deuterium (D8) labelled arachidonic acid 5 days PF. Systemic injection of D8-labelled arachidonic acid resulted in very high HDF activity in Challenged mosquitoes, but not in Naive controls (Fig. 3a; Supplementary Fig. 8). The biological activity was robust even after diluting the haemolymph from Challenged mosquitoes 100-fold before the transfer (Supplementary Fig. 8). Liquid chromatography tandem MS (LC-MS/MS)-based LM metabololipidomics confirmed the production of d8-LXA_4_ and d8-LXB_4_ at higher levels in haemolymph of Challenged mosquitoes (Fig. 3b–f). No other LMs were found. In general, LXA_4_ synthesis was lower than LXB_4_ in the Naive group (Fig. 3c). However, the relative increase in conversion following an immune challenge was very high for LXA_4_ (100-fold, *P*<0.01, Fig. 3d), while the increase in LXB_4_ synthesis was more modest and variable and did not reach statistical significance. This indicates that mosquitoes have the capacity to convert precursor arachidonic acid to lipoxins, and that immune challenge results in a sustained increase in lipoxin synthesis, predominantly LXA_4_.

**Figure 4:**
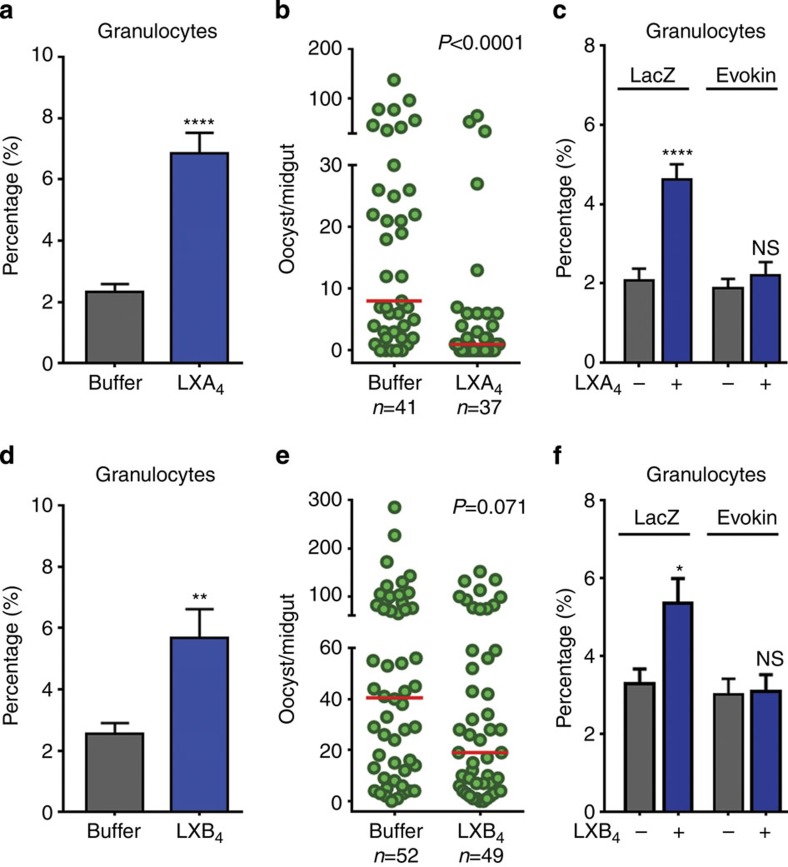
Biological activity of systemic injection of synthetic lipoxins Effect of (**a**) LXA_4_ or (**d**) LXB_4_ on granulocytes and of (**b**) LXA_4_ or (**e**) LXB_4_ on *P. berghei* infection. Effect of Evokin silencing on the biological activity of (**c**) LXA_4_ and (**f**) LXB_4_. Error bars in **a**,**c**,**d** and **f** represent mean±s.e.m. Granulocyte proportions were determined for each individual mosquito. Haemocytes were counted in 6–10 mosquitoes for each treatment and the results were confirmed in at least 2 independent experiments. Each circle in **b** and **e** represents the number of parasites in an individual midgut and the line indicates the medians. Results represent data from at least two independent experiments. Mann–Whitney test, *P≤0.05; **P≤0.01; ****P≤0.0001; NS, *P*>0.05.

The biological activity of these two candidate LMs was tested by injecting mosquitoes with chemically synthesized LXA_4_ and LXB_4_. We tested the biological activity in a dose–response curve and obtained a response for LXA_4_ at doses between 5 and 500 nM and between 50 and 500 nM for LXB_4_ (Supplementary Fig. 9). Administration of either LM recapitulated the activity of HDF, increasing the number of circulating granulocytes (Fig. 4a,d) and enhancing antiplasmodial immunity (Fig. 4b,e), with the response to LXA_4_ being more robust (Fig. 4a,d; Supplementary Fig. 10). Injection of 4-HDHA had no effect on haemocyte differentiation nor immunity to *Plasmodium* (Supplementary Fig. 11).

### Functional interaction between evokin and lipoxins

Finally, the importance of Evokin as a lipoxin carrier was tested by silencing expression before the administration of synthetic LXA_4_ or LXB_4._ The biological activity of LXA_4_ and LXB_4_ was completely eliminated when Evokin was silenced, confirming the likely role of this lipocalin carrier (Fig. 4c,f) in protecting lipoxins from degradation. Interestingly, vertebrate ApoD has been found to bind arachidonic acid with high affinity^8^,^9^,^12^.

## Discussion

Specific eicosanoids modulate insect haemocytes responses such as migration, phagocytosis, microaggregation, nodulation, spreading and melanization, at nano and picomolar concentrations, but very few mediators have been characterized^13^,^14^,^15^,^16^,^17^. Most insect studies describe the effect of administrating eicosanoids or drugs that inhibit prostaglandin synthesis. However, the biological relevance of eicosanoids in insect immunity has been difficult to demonstrate because no orthologues of lipoxygenases or cyclooxygenases are apparent in their genomes^13^. In *Drosophila*, disruption of a haeme peroxides gene (*ptx*) results in defects in ovarian follicle maturation and female sterility that can be restored by expressing mammalian Cox1 protein, indicating that Ptx is the insect orthologue of Cox1 (ref. 18). Lipoxins are biosynthesized via lipoxygenases and have been extensively studied in vertebrates^19^,^20^, but have not been reported in insects. We envision that immune challenge results in constitutive expression of the enzymes mediating lipoxin biosynthesis, similar to what we observed for Evokin, probably through an epigenetic mechanism. The nature of these enzymes in mosquitoes or any other insect remains to be defined. We have previously shown that disruption of the four signalling pathways known to mediate antiplasmodial immunity (*STAT*, *JNK, Toll* and IMD) pathways did not disrupt HDF production^7^. However, the *STAT, JNK* and *Toll* pathways are required for haemocytes to respond to HDF^7^. The signalling pathway(s) that mediate the establishment of immune priming are not known, but we hypothesize that a signal from the midgut epithelium is inducing a long-lasting increase of Evokin in the mosquito body wall.

Within vertebrates, lipoxins have potent anti-inflammatory and pro-resolution properties, stimulating non-inflammatory monocyte recruitment, macrophage phagocytosis of microbes and cellular debris, and increased chemotaxis and laminin adhesion^16^,^19^,^21^,^22^,^23^,^24^,^25^. The role of lipoxins in *A. gambiae* immune priming described here raises the question of whether, in addition to promoting resolution of an infectious process, lipoxins may also prepare vertebrate hosts for subsequent challenges by priming cells of the innate immune system.

## Methods

### Mosquito maintenance and *P. berghei* infection

*A. gambiae* (G3 strain) mosquitoes were reared in standard insectary conditions at 28 ^o^C, 80% humidity, with a 12-h light/dark cycle and maintained with 10% Karo syrup solution. Infections with *P. berghei* were conducted using the *P. berghei* green fluorescent protein–CON transgenic 259cl2 strain. Mouse infections were maintained through serial passage in 3–4-week-old female BALB/c mice and parasitemias were evaluated by light microscopy from blood smears that were methanol fixed, stained with 10% Giemsa and air-dried prior to visualization. Mosquito infections were done by allowing mosquitoes to feed on green fluorescent protein *P. berghei* mouse that had a 3–5% parasitemia and with one to two exflagellations per field. Mosquitoes were then maintained at 19 ^o^C, 80% humidity and a 12-h light/dark cycle until the day of haemolymph collection or midgut dissection. To determine the intensity of infection, mosquitoes were dissected at 7 days PF and oocyst counted in individual midguts. Results were confirmed in at least two independent experiments.

### Ethics statement

Public Health Service Animal Welfare Assurance #A4149-01 guidelines were followed according to the National Institutes of Health Animal (NIH) Office of Animal Care and Use (OACU). These studies were done according to the NIH animal study protocol (ASP) approved by the NIH Animal Care and User Committee (ACUC), with approval ID ASP-LMVR5.

### Haemolymph collection and haemocyte counting

Haemolymph was collected as previously described^2^. In short, to count haemocytes, a very fine incision (about half a millimeter) was made in the mosquito between the last two abdominal segments using sterile fine-tip forceps and then injected with an anticoagulant solution containing 60% Schneider’s Insect medium, 10% foetal bovine serum and 30% citrate buffer. Enough anticoagulant buffer was injected until 10 μl was recovered using a sterile pipette tip from the incision at the end of the abdomen. For further biochemical characterization, haemolymph was stored at −80 ^o^C until use. For haemocyte counting, the collected sample was deposited in a sterile disposable haemocytometer slide (10 μl capacity, Neubauer Improved, iNCYTO C-Chip DHC-N01) and the haemocytes counted using a light microscope ( × 40 objective), distinguishing granulocytes from the rest of the haemocyte types (prohaemocytes and oenocytoids)^2^. The total number of haemocyte cells and the proportion of granulocytes were determined for each individual mosquito. Haemocytes were counted in 6–10 in mosquitoes for each treatment and the results were confirmed in at least 2 independent experiments.

### Haemolymph transfer

Transfers of cell-free haemolymph were conducted as previously described^2^,^6^. Briefly, haemolymph was collected by perfusion, as described above, from about 50 Naive and 50 Challenged mosquitoes at 7 days post priming using a modified anticoagulant buffer (95% Scheider’s medium and 5% citrate buffer). Following centrifugation at 4 ^o^C, 9,300*g* for 10 min, the cell-free supernatant was transferred to a new microcentrifuge tube, aliquoted and stored at −80 ^o^C until use. Transfer into sugar-fed Naive mosquitoes was done by microinjection of a total of 138 nl per mosquito. We transferred the equivalent of one-twentieth of the haemolymph from the mosquito donors to the recipients. The effect of haemolymph transfer on the proportion of granulocytes in the recipient was evaluated 4 days post injection. To evaluate the effect of transfer on *Plasmodium* infection, recipient mosquitoes were fed on a *P. berghei*-infected mouse 3 days post injection and the number of oocysts in individual midguts was determined 7 days post feeding.

To assess the effect of Evokin (AGAP009281) silencing on HDF bioactivity, 2–3-day-old donor mosquitoes were injected with double stranded RNA (dsRNA) for LacZ (control) or Evokin and fed on an infected or uninfected mouse at 3 days post injection. Mosquitoes were maintained at 19 ^o^C for 48 h then transferred to 28 ^o^C with haemolymph collected 7 days post-blood feeding. Haemolymph transfer to Naive recipients was done by injecting 138 nl of cell-free haemolymph from LacZ or Evokin-silenced donors.

### Gene expression analysis

For gene expression analyses, midguts and abdominal fat body walls were collected at 24 h and 7 days post-blood meal. Midguts were homogenized and processed for RNA using the RNeasy RNA extraction kit (Qiagen) according to manufacturer’s instructions. Fat bodies, in turn, were homogenized in TRIzol and processed for RNA according to the manufacturer/s instructions. The quality and concentration from each RNA sample was assessed via NanoDrop (Thermo Scientific) and the same amount of RNA was used for complementary DNA (cDNA) synthesis in a given experiment. Synthesis of cDNA was performed using the QuantiTect reverse transcription kit with DNA wipeout (Qiagen). The resulting cDNA was then used as a template for subsequent relative quantification via quantitative PCR (qPCR) using the DyNamo SYBR green qPCR kit (Thermo Scientific) with target specific primers (Supplementary Table 1). For each experiment, the quantitative reverse transcription–PCR messenger RNA expression analysis was done in two groups of midguts or body walls dissected from 15 mosquitoes (biological replicates) that were analysed in duplicate (technical replicates) and the reproducibility of the result was confirmed in three independent experiments. The assay was run on a CFX96 Real-Time PCR Detection System (Bio-Rad) and data analysed post run using the ΔΔCt method. The statistical significance of fold change values was determined using the Mann–Whitney test (GraphPad, La Jolla, California, USA).

### dsRNA synthesis and gene silencing

The RNA interference (RNAi)-directed silencing of Evokin was performed as previously described^26^. Briefly, 2–3-day-old female *A. gambiae* were cold-anesthetized and injected with 69 nl of a 3 μg per μl dsRNA solution. Synthesis of dsRNA was generated using a cDNA template from *A. gambiae* and the MEGAscript RNAi kit (Ambion). Primers were designed to specifically target the Evokin transcript and are listed in Supplementary Table S1. The dsRNA-injected mosquitoes were then used in subsequent assays at 3 days post injection. The silencing efficiency attained through this method was 80% for both midgut and fat body tissues relative to dsLacZ controls.

### Heat treatment of HDF

Haemolymph supernatants were heated at 70 ^o^C for 30 min then incubated on ice for an additional 30 min. Samples were then centrifuged at 4 ^o^C, 9,300*g* for 30 min. The supernatant was then removed and stored at −20 ^o^C until use.

### HDF ethanol precipitation

The collected haemolymph supernatant was brought to 90% ethanol by adding ethanol at a 1:9 (v:v) proportion, and incubated on ice for 1–2 h. The sample was then centrifuged at 4 ^o^C, 9,300*g* for 10 min, transferred to a new microcentrifuge tube and the ethanol evaporated using a speed vac. The dried sample was washed with Milli-Q water and dried once more and then re-suspended in molecular grade water or transfer buffer by vortexing for 30–60 min at 4 ^o^C. The pH was measured with a pH meter strip and adjusted to pH 7; the sample was stored at −20 ^o^C until use.

### HDF heat treatment and ethanol precipitation

The haemolymph supernatant was heat treated as described above, brought to 90% ethanol by adding ethanol at a 1:9 (v:v) proportion, and incubated on ice for 1–2 h. The sample was then centrifuged at 4 ^o^C, 9,300*g* for 10 min, the supernatant transferred to a new tube and the ethanol evaporated using a speed vac. The dried samples were re-suspended in Milli-Q water (original haemolymph supernatant volume) and dried one more time before re-suspension in molecular grade water or transfer buffer. The pH was measured with a pH meter strip and adjusted to pH 7; the sample was stored at −20 ^o^C until use.

### HDF proteinase K treatment

The haemolymph supernatant was mixed with proteinase K at a final concentration of 100 μg ml^−1^ and incubated at 60 ^o^C for 1 h. The enzyme was then inactivated by heating at 80 ^o^C for 30 min. The sample was centrifuged at 4 ^o^C, 9,300*g* for 10 min and the supernatant stored at −20 ^o^C until use.

### Lipid extraction

Lipid extractions were carried out following the Bligh and Dyer^27^ method with slight modifications. Briefly, haemolymph was placed in a 5 ml glass tube (Kimble Chase) and mixed with chloroform and methanol (0.8:1:2, v-v:v). The mixture was mixed by vortexing every 10 min for 1 h and then centrifuged at 580*g* for 10 min at room temperature. The supernatant was transferred to a new glass tube and mixed with an additional 0.5 ml of Milli-Q water and 0.5 ml of chloroform and vigorously mixed in using a vortex. The mixture was centrifuged again at 580*g* for 10 min at room temperature to obtain a uniform phase separation. Both aqueous and organic phase were separately transferred to a 5 ml glass tube and taken to dryness with nitrogen gas. Dried samples were either re-suspended in water and used immediately or re-suspended in 100% methanol for storage at −80 ^o^C.

### Reverse-phase (C18) separation

Lipid extracted samples were further separated according to their polarity using a pipette tip column of C18 resin (TARGA Reversed Phase, UltraMicroSpin, The Nest Group, Inc., Southborough, MA). The C18 spin columns were first conditioned according to manufacturer’s instructions, loaded with either the aqueous or the re-suspended organic phase, spun or forced with a syringe and then washed once with HPLC water prior to elution. Elution was done by first eluting with 100 μl of HPLC-grade methanol, followed by 100 μl of HPLC-grade methyl acetate and finally with 100 μl of HPLC-grade hexane. The resultant eluate were dried under nitrogen gas, re-suspended in 100% methanol and stored at −80 ^o^C until use.

### Normal phase HILIC fractionation using a microtip column

The HILIC tip columns were first conditioned according to manufacturer’s instructions (The Nest Group, Inc.). Prior to applying the sample into a conditioned column, the haemolymph sample was brought to a 85% acetonitrile solution (final pH 6-7) with a total volume of 100 μl and loaded into the tip column. The tip column was centrifuged at 180*g* for 2 min, washed once with 100 μl of 85% acetonitrile and both flow-through were combined (FT fraction). The sample was then eluted with decreasing concentrations of acetonitrile (80, 40, 20, 10 and 5% acetonitrile in water). Each elution was done with 100 μl of elution buffer and centrifuged at 180*g* for 1–3 min. The eluted fractions were then dried under speed vac, and washed three times with 40 μl of water. Finally the dried fractions were re-suspended in 20 μl of water and vortexed in the cold room for 30–60 min. The pH of each fraction was measured, adjusted to pH 7.0 and stored at −20 ^o^C until used.

### Deuterium-labelled arachidonic acid injections

Four- to 5-day-old mosquitoes were allowed to feed on either an infected or uninfected mouse, maintained at 19 ^o^C for 3 days, then transferred at 27 ^o^C and provided with oviposition cups. Five days post feeding 50–80 mosquitoes were injected with 1 μg of deuterated arachidonic acid (arachidonic acid-d8) re-suspended in 1% BSA in Hanks Balanced Salt Solution (without Ca^+2^ and Mg^+2^). Haemolymph was then collected via perfusion from uninfected mosquitoes (Naive) and infected mosquitoes (Challenged) at 24 h post injection. Prior to lipid extraction and C18 fractionation, both Naive and Challenged samples were spiked with 400 pg of Resolvin D2-d_5_ as internal standard for lipid extraction efficiency. Processed samples were then stored in amber-glass vials at −80^o^C until needed.

### Eicosanoid injection

Two- to 3-day-old *A. gambiae* mosquitoes were cold-anesthetized and injected with serial dilutions of either Lipoxin A_4_ (0.5–5,000 nM) or Lipoxin B_4_ (0.5–500 nM). The maximum response was obtained after an estimated initial concentration in mosquito haemolymph of 500 nM (Supplementary Fig. 9), and this dose was used in all subsequent experiments. Lipoxins were dried using a stream of nitrogen gas and suspended in modified Schneider’s medium (devoid of amino acids and containing 5% citrate buffer). Mosquitoes were either fed on a *P. berghei*-infected mouse at 3 days post injection to assess its effect on *Plasmodium* infection, or perfused 4 days post injection to assess the proportion of granulocytes.

### HILIC–HPLC

Pooled haemolymph from 400–500 Naive or Challenged mosquitoes were loaded onto a hydrophilic interaction column (PolyHYDROXYETHYL A, 100 × 2.1 mm ID, 300-Å pore, PolyLC Inc., Columbia, MD) equilibrated with 90% of acetonitrile (LC/MS quality acetonitrile, acid free, Fisher Scientific) using the AKTA purifier system (GE Healthcare, Piscataway, NJ). Fractions were eluted with a linear gradient of 90-0% acetonitrile in water over 30 min at a flow rate of 0.25 ml min^−1^. Absorption of the eluate was monitored at 225 and 280 nm. Bioactive fractions were dried under vacuum and sent for LC/MS analysis using an Agilent 1,100 nanoflow LC system (Agilent Technologies, Palo Alto, CA) coupled online with a linear ion-trap mass spectrometer (LTQ, ThermoElectron, San José, CA). Nanoflow reverse-phase LC-MS/MS was performed as described before^28^. Tandem mass spectra were searched using SEQUEST on a 20-node Beowulf cluster against the NR NCBI proteome database with methionine oxidation included as dynamic modification.

### LM metabololipidomics

Fraction from Naive and Challenged mosquitoes were isolated as outlined above and subjected to LM metabololipidomics as in the study by Colas *et al.*^29^, with some modifications. An Agilent Poroshell 120 EC-C18 column (100 × 4.6 mm × 2.7 μm) was kept at 50 °C and LM was eluted with a mobile phase consisting of methanol/water/acetic acid of 50:50:0.01 (v:v:v) that was ramped to 80:20:0.01 (v:v:v) from 2 to 11 min, maintained till 14.5 min and then rapidly ramped to 98:2:0.01 (v:v:v) for the next 0.1 min. This was maintained at 98:2:0.01 (v:v:v) for 5.4 min, and the flow rate was maintained at 0.5 ml min^−1^. Deuterated (d)_5_-RvD2 was employed to facilitate sample recovery and quantification. To monitor d_8_-LXA_4_ and d_8_-LXB_4_ in fractions isolated from d_8_-AA treated mosquitoes, an multiple reaction monitoring method was developed using the following transitions 359.1>116.1 and 359.1>227.1, respectively. Absolute quantities for lipoxin synthesis were derived from LC-MS/MS lipidomics data obtained from two independent pools of mosquito haemolymph (50–80 mosquitoes per pool). The abundance of lipoxins in the Challenged groups (relative to the Naive controls) was calculated by dividing d_8_-LXA_4_ or d_8_-LXB_4_ levels in Naive and Challenged groups by the average d_8_-LXA_4_ or d_8_-LXB_4_ levels in the Naive samples.

### Statistical analysis

All statistical analyses were performed using GraphPad Prism 5 (GraphPad). All analyses where pertinent were conducted using either the student unpaired *t*-test or the Mann–Whitney test. Significance was assessed at *P*<0.05. The error bars represent the s.e.m.

## Additional information

**How to cite this article:** Ramirez, J. L. *et al.* A mosquito lipoxin/lipocalin complex mediates innate immune priming in *Anopheles gambiae*. *Nat. Commun.* 6:7403 doi: 10.1038/ncomms8403 (2015).

## Supplementary Material

Supplementary Information Supplementary Figures 1-11, and Supplementary Table 1
