# Author Correction: A mosquito lipoxin/lipocalin complex mediates innate immune priming in *Anopheles gambiae*

**DOI:** 10.1038/s41467-023-39932-1

**Published:** 2023-07-18

**Authors:** Jose Luis Ramirez, Giselle de Almeida Oliveira, Eric Calvo, Jesmond Dalli, Romain A. Colas, Charles N. Serhan, Jose M. Ribeiro, Carolina Barillas-Mury

**Affiliations:** 1 Laboratory of Malaria and Vector Research, National Institute of Allergy and Infectious Diseases, National Institutes of Health, Rockville, MD 20852 USA; 2 Center for Experimental Therapeutics and Reperfusion Injury, Department of Anesthesiology, Perioperative, and Pain Medicine, Brigham and Women’s Hospital and Harvard Medical School, Boston, MA 02115 USA; 3 Present Address: Laboratório de Entomologia Médica, Centro de Pesquisas Rene Rachou—Fiocruz, Belo Horizonte, Brazil

Correction to: *Nature Communications* 10.1038/ncomms8403, published online 23 June 2015

The original version of this Article contained an error in Figure 3 legend, which failed to indicate that panel b is an illustration derived, by not directly reproduced, from the original mass spectrum output. The correct Figure 3b legend should read “Illustrations derived from the chromatographs obtained by Multiple Reaction Monitoring (MRM) of the parent ion (Q1) m/z 359 and a diagnostic daughter ion (Q3) for deuterium-labelled (d8) LXA4 and LXB4”. This has been corrected in both the PDF and HTML versions of the Article. Additional, supplementary mass spectrum method information is also appended to the updated Supplementary Information file to assist in the interpretation of data in Figure 3.

## Supplementary information

Updated Supplementary Information

