# Supplementary material for: A mosquito lipoxin/lipocalin complex mediates innate immune priming in Anopheles gambiae
Source: Nat Commun. 2015 Jun 23;6:7403. doi: 10.1038/ncomms8403 (PMC4542143; doi:10.1038/ncomms8403)
Supplement: Supplementary Information — Supplementary Figures 1-11, and Supplementary Table 1 [file ncomms8403-s1.pdf]

SUPPLEMENTARY TABLE AND FIGURES

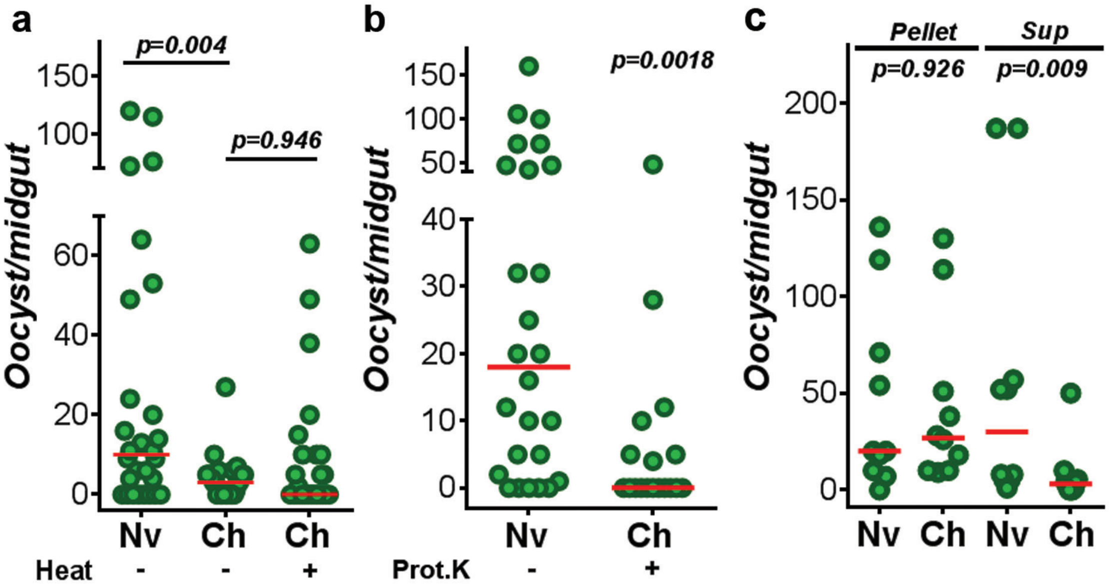

Supplementary Figure 1. **Biochemical characterization of HDF**. **(a)** Effect of Heat-treatment and **(b)** Proteinase K (Prot.K) on HDF biological activity determined by its ability to enhance antiplasmodial immunity in recipient mosquitoes. **(c)** Effect of Heat-treatment followed by ethanol precipitation on HDF biological activity determined by the ability to enhance antiplasmodial immunity in recipient mosquitoes. Nv: Naïve. Ch: Challenged. Sup: Supernatant. Nv: Naïve. Ch : Challenged. Each circle represents the number of parasites in an individual midgut and the red line indicates the medians. Mann-Whitney test, *, p ≤ 0.05; **, p ≤ 0.01; ***, p ≤ 0.001; ****, p ≤ 0.0001

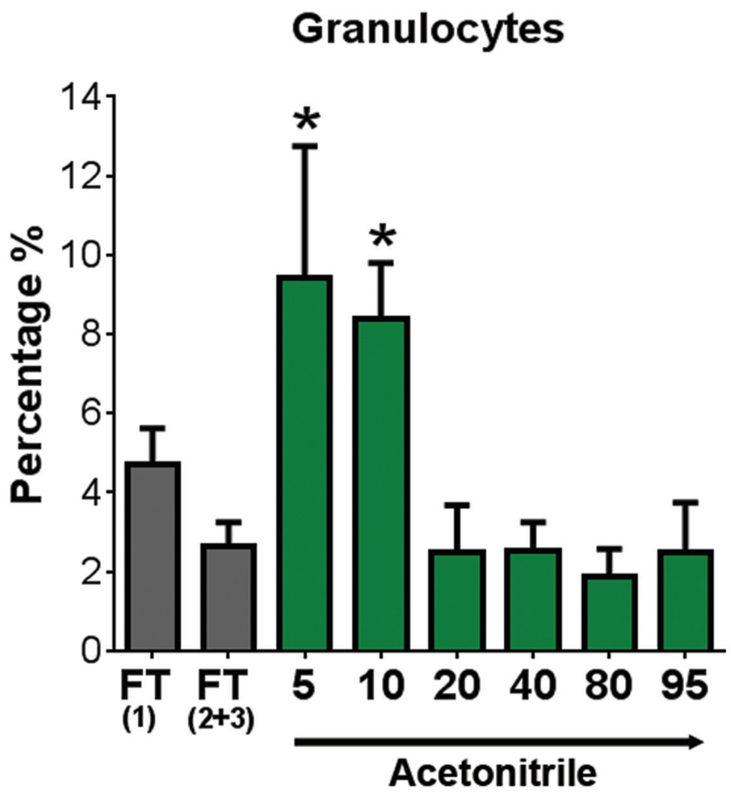

Supplementary Figure 2. HDF bioactivity in fractions after step-elution of a reversed-phase (C18) TARGA Minispin column with increasing concentrations of acetonitrile. HDF biological activity was determined by the ability to increase the percentage of granulocytes in recipient mosquitoes. FT1 = Flow-through and FT2+3 = the two subsequent washes with water prior to elution with acetonitrile. Error bars represent mean ± SEM. Granulocyte proportions were determined for each individual mosquito. Hemocytes were counted in 6 – 10 mosquitoes for each treatment and the results were confirmed in at least two independent experiments. Mann-Whitney test, *, $p \leq 0.05$.

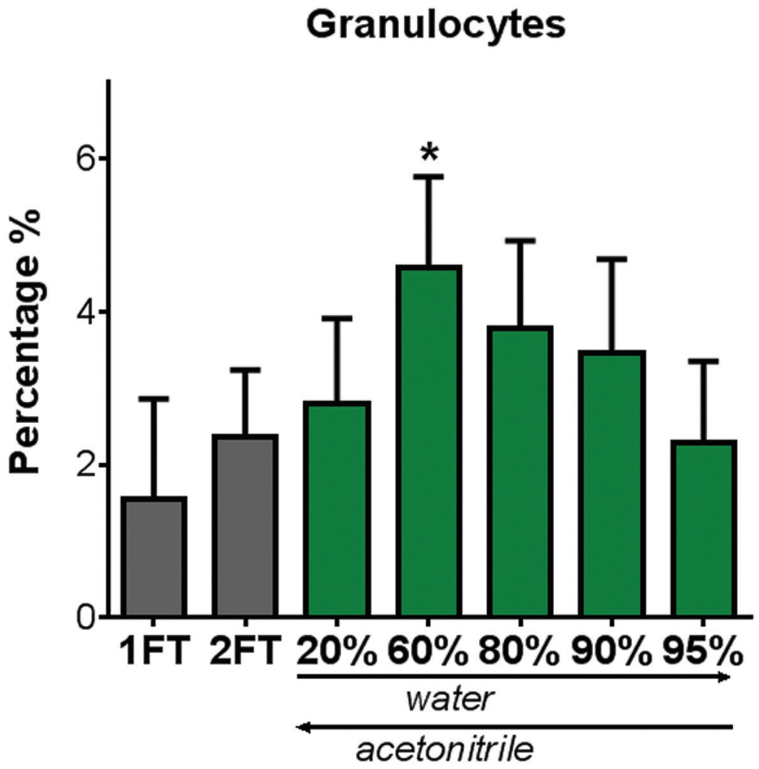

Supplementary Figure 3. HDF bioactivity in fractions after step-elution of a HILIC microtip column with increasing concentrations of water. HDF biological activity was determined by the ability to increase the percentage of granulocytes in recipient mosquitoes. 1FT= Flow-through and 2FT = subsequent wash with acetonitrile prior to elution with increasing concentrations of water. Error bars represent mean ± SEM. Granulocyte proportions were determined for each individual mosquito. Hemocytes were counted in 6 – 10 mosquitoes for each treatment and the results were confirmed in at least two independent experiments. Mann-Whitney test, *, $p \leq 0.05$.

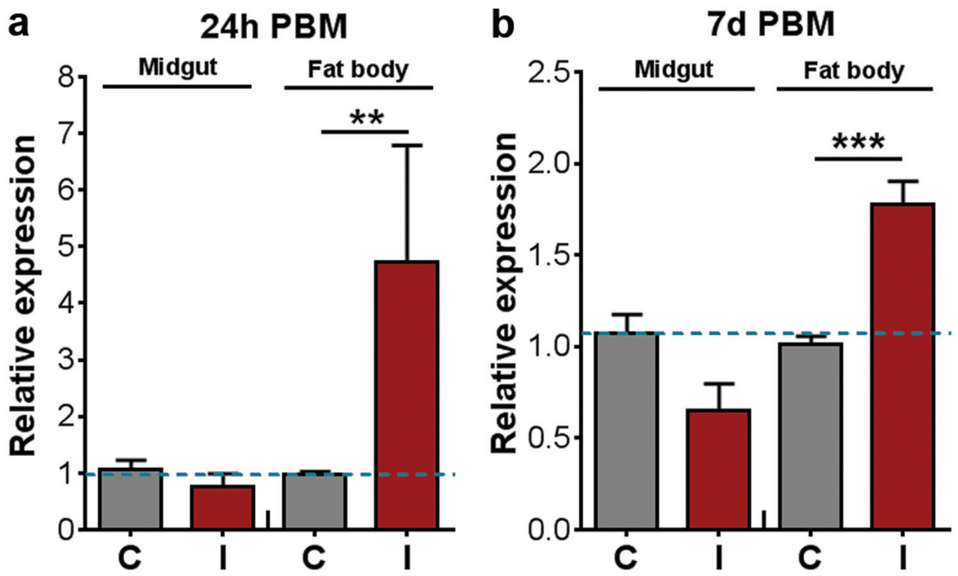

Supplementary Figure 4 . Evokin mRNA expression in the mosquito midgut and fat body following *P. berghei* infection. Evokin relative expression in the midgut and abdominal fat body at **(a)** 24 hours and **(b)** 7 days post-blood meal (PBM) on an control or *P. berghei*-infected mouse. Dotted blue line indicates basal levels of Evokin expression found in the control group. C: control; I: Infected. qRT-PCR mRNA expression analysis was done in two groups of midguts or body walls dissected from 15 mosquitoes (biological replicates) and analyzed in duplicate (technical replicates). The reproducibility of the result was confirmed in three independent experiments. Error bars represent mean ± SEM. Mann-Whitney test, **, $p \leq 0.01$; ***, $p \leq 0.001$.

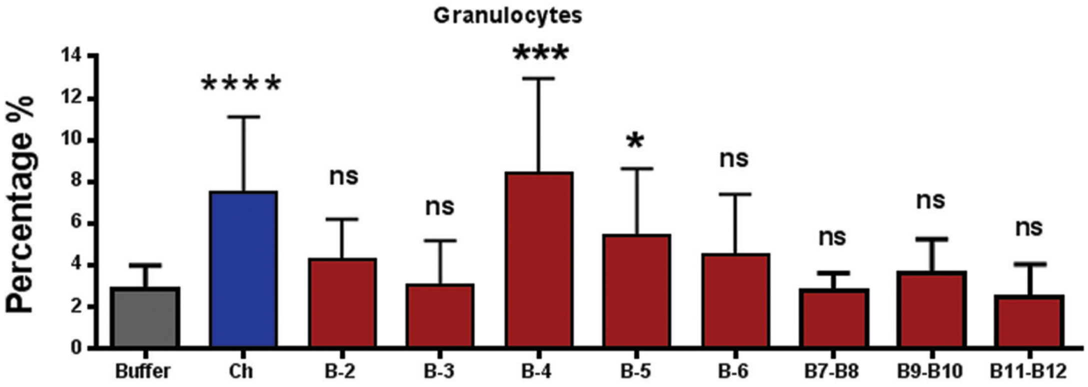

Supplementary Figure 5. HDF bioactivity of challenged hemolymph HILIC chromatography fractions. HDF biological activity was determined by the ability to increase the percentage of granulocytes in recipient mosquitoes, Activity in hemolymph from challenged mosquitoes (blue bar) and HILIC fractions (red bars). Injection of buffer alone was used as negative control (grey bar). Ch: Challenged. Error bars represent mean ± SEM. Granulocyte proportions were determined for each individual mosquito. Hemocytes were counted in 6 – 10 mosquitoes for each treatment and the results were confirmed in at least two independent experiments. Mann-Whitney test, *, $p \leq 0.05$; **, $p \leq 0.01$; ***, $p \leq 0.001$; ****, $p \leq 0.0001$; ns, $p>0.05$

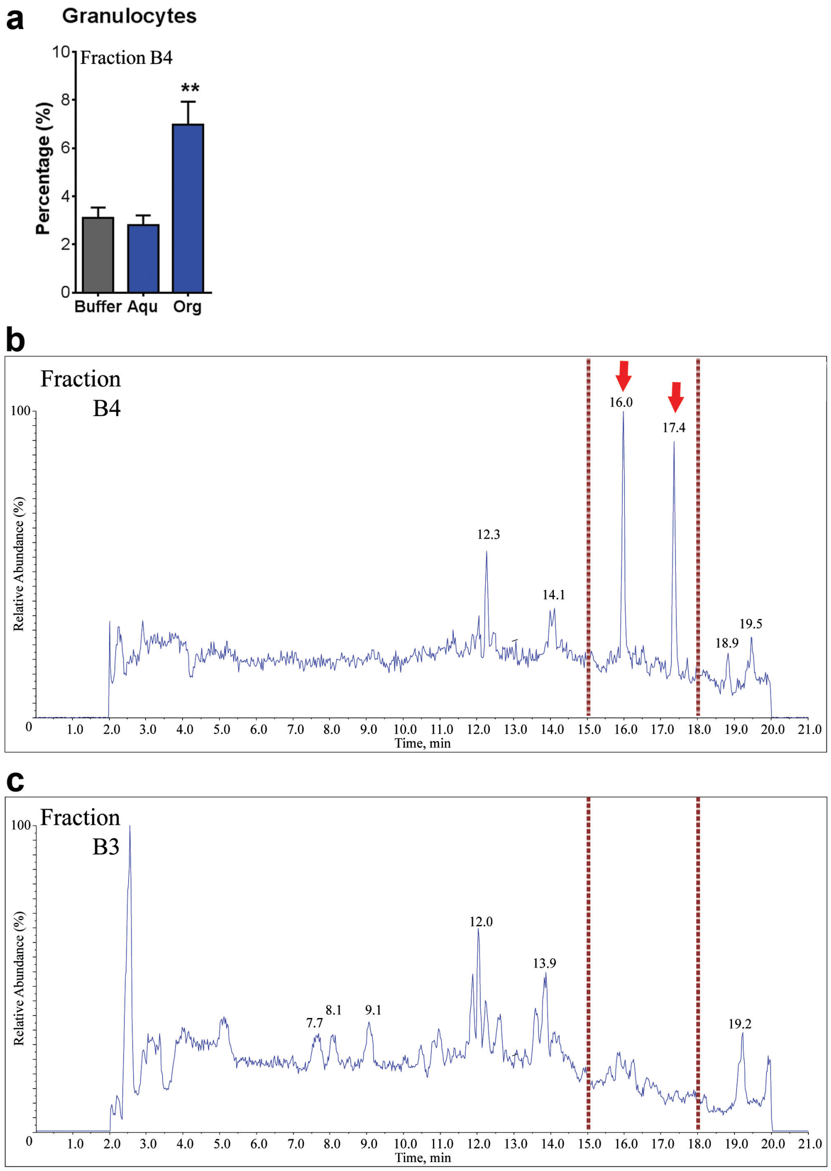

Supplementary Figure 6. HDF bioactivity and lipid mediator lipidomic (a) HDF bioactivity of HILIC fraction B4 following lipid extraction (Aqu: Aqueous phase; Org: Organic phase). Lipid mediator lipidomic chromatographs of **(b)** HILIC fraction B4 (highest HDF activity), and of **(c)** HILIC fraction B3 (lacking HDF activity). Red arrows indicate the two prominent peaks present in the fraction with HDF activity (organic phase of fraction B4). Error bars represent mean ± SEM. Granulocyte proportions were determined for each individual mosquito. Hemocytes were counted in 6 – 10 mosquitoes for each treatment and the results were confirmed in at least two independent experiments. Mann-Whitney test, **, $p \leq 0.01$

a

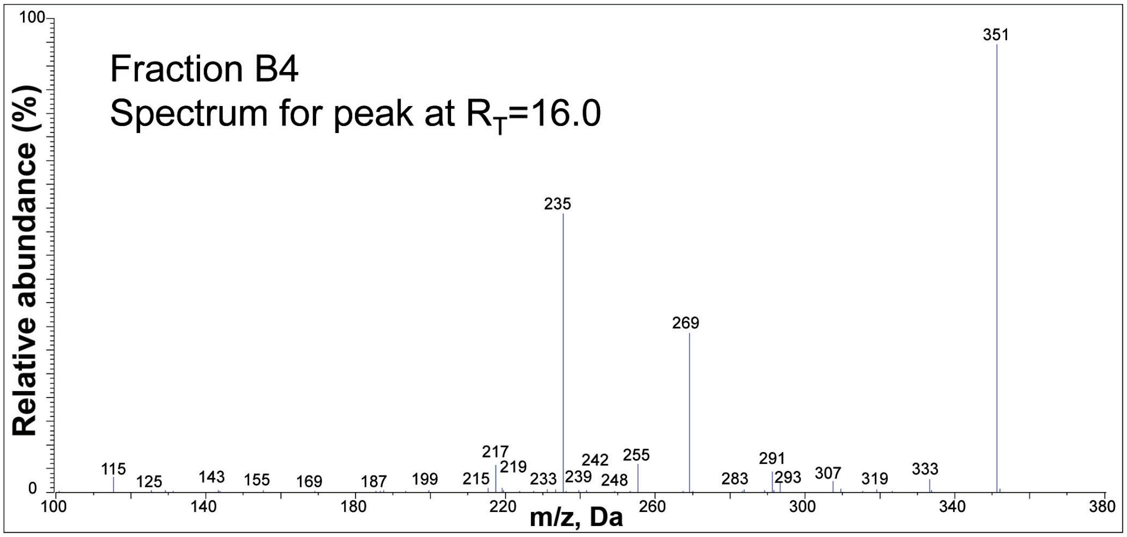

b

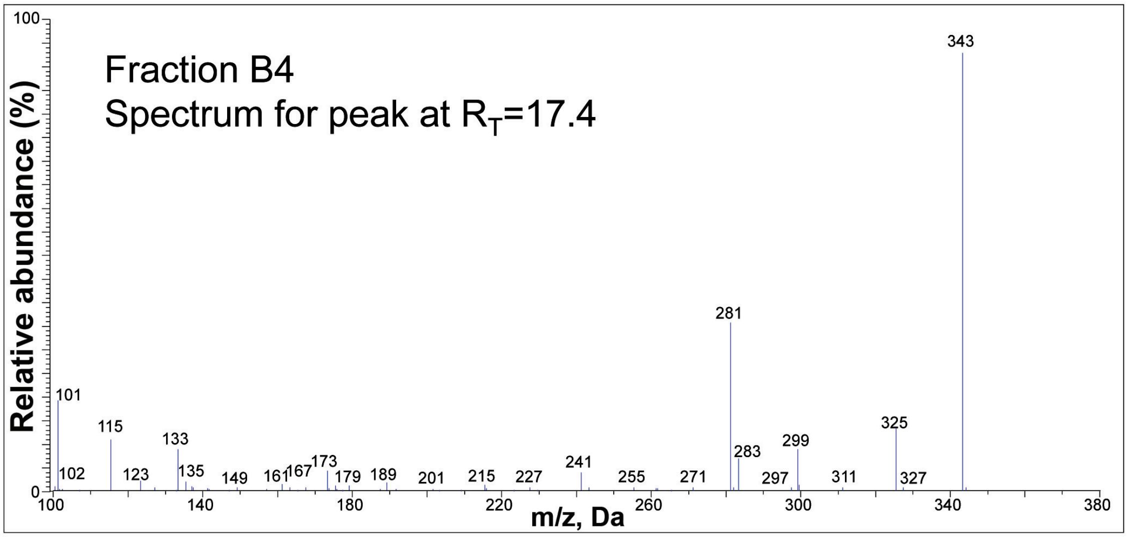

Supplementary Figure 7. LC MS/MS spectra of the two prominent peaks observed in fraction B4. (a) MS/MS spectrum of peak at Rt=16,0 ; a compound that shares mass spectrum signatures with vertebrate Lipoxins. **(b)** MS/MS spectrum of peak at Rt=17.4 ; a compound that shares mass spectrum signatures with 4-hydroxy-docosahexaenoic acid (4-HDHA).

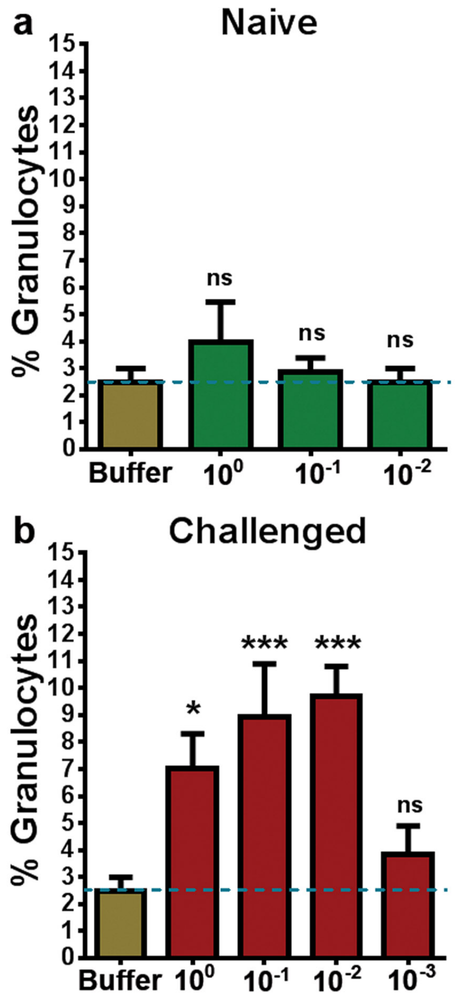

Supplementary Figure 8. Effect of systemic injection of deuterium-labeled arachidonic acid on HDF biological activity. Graphs represent the proportion of granulocytes in recipient mosquitoes in response to injection of different hemolymph concentrations (10-fold dilutions) from **(a)** naïve and **(b)** challenged donors. Donors were injected with d8-arachidonic acid 5 days post-feeding and hemolymph was collected 24h post-injection. Mosquitoes injected with buffer served as controls. Error bars represent mean ± SEM. Granulocyte proportions were determined for each individual mosquito. Hemocytes were counted in 6 – 10 mosquitoes for each treatment and the results were confirmed in at least two independent experiments. Mann-Whitney test, *, $p \leq 0.05$; **, $p \leq 0.01$; ***, $p \leq 0.001$; ns, $p>0.05$

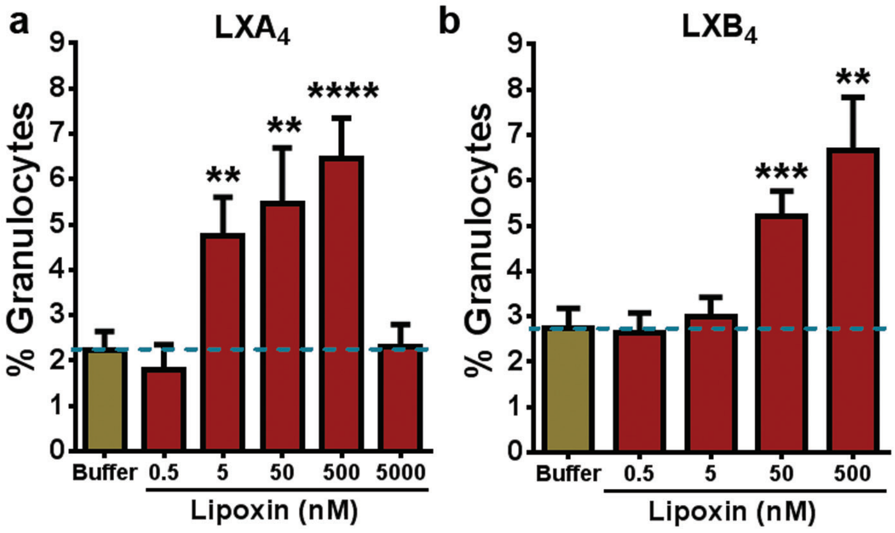

Supplementary Figure 9. Effect of systemic injection of synthetic Lipoxins on the proportion of granulocytes. Graphs represent the proportion of granulocytes in response to injection of different concentrations (10-fold dilutions) of **(a)** Lipoxin A_4 and **(b)** Lipoxin B_4. Mosquitoes injected with buffer served as controls and perfusions for hemocyte counting were done 4 days post-injection. Error bars represent mean ± SEM. Granulocyte proportions were determined for each individual mosquito. Hemocytes were counted in 6 – 10 mosquitoes for each treatment and the results were confirmed in at least two independent experiments. Mann-Whitney test, **, $p \leq 0.01$; ***, $p \leq 0.001$; ****, $p \leq 0.0001$.

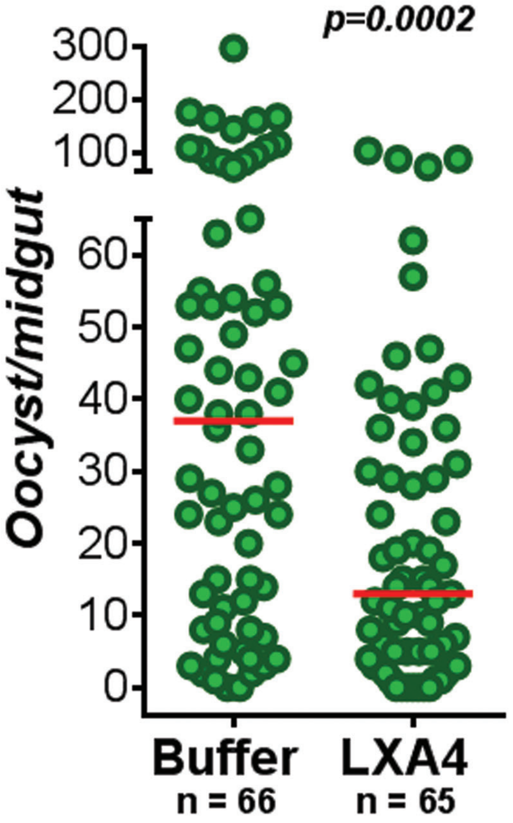

Supplementary Figure 10. Effect of synthetic Lipoxin A4 injection on *Plasmodium berghei* infection. Graph represents data from two independent feedings. Red horizontal line represents the median oocyst per midgut in buffer-injected (control) or LXA4-injected mosquitoes. Each circle represents the number of parasites in an individual midgut and the line indicates the medians. Mann-Whitney test, ***; p=0.0002

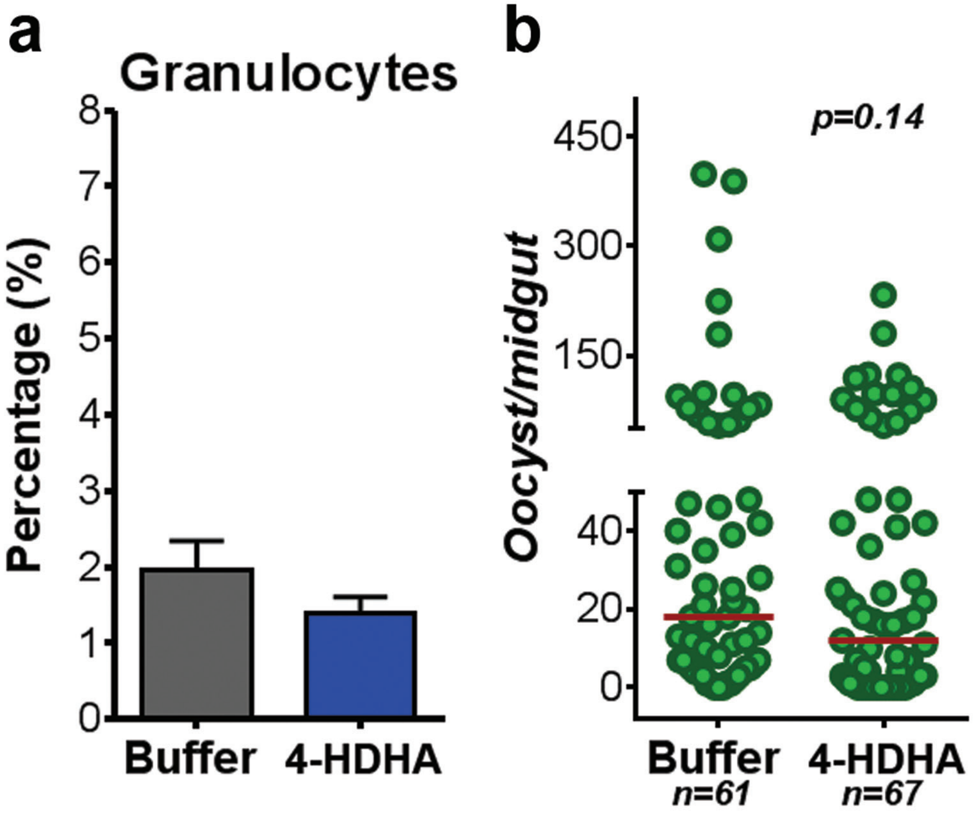

Supplementary Figure 11. Effect of synthetic 4-HDHA injection on (a) proportion of granulocytes and on **(b)** *Plasmodium berghei* infection. Graph represents data from two independent infections. Red horizontal line represents the median oocyst per midgut in buffer-injected (control) or 4-HDHA-injected mosquitoes. Error bars in fig. a represent mean ± SEM. Granulocyte proportions were determined for each individual mosquito. Hemocytes were counted in 6 – 10 mosquitoes for each treatment and the results were confirmed in at least two independent experiments. Each circle in figure b represents the number of parasites in an individual midgut and the line indicates the medians. Mann-Whitney test, ns, $p \geq 0.05$

Supplementary Table 1. Primer sequences used for Evokin silencing, gene expression analysis, and assessment of silencing efficiency.

Primer name	Primer sequence	AGAP -ID
**dsAG9281-F**	taatacgactcactatagggTGGCGGTACTCCTGCTACTC*	AGAP009281
**dsAG9281-R**	taatacgactcactatagggGCCGTTATACACTTCCCACC	AGAP009281
**Eff-2-9281-F**	CAGCCGAAAGTGAACAAACA	AGAP009281
**Eff-2-9281-R**	ATGGTGGCCATTGTATACGG	AGAP009281

*T7 promoter sequence is indicated in lower case letters.
