# Supplementary material for: Author Correction: A mosquito lipoxin/lipocalin complex mediates innate immune priming in Anopheles gambiae
Source: Nat Commun. 2023 Jul 18;14:4305. doi: 10.1038/s41467-023-39932-1 (PMC10354017; doi:10.1038/s41467-023-39932-1)
Supplement: Supplementary file 1 — Updated Supplementary Information [file 41467_2023_39932_MOESM1_ESM.pdf]

SUPPLEMENTARY TABLE AND FIGURES

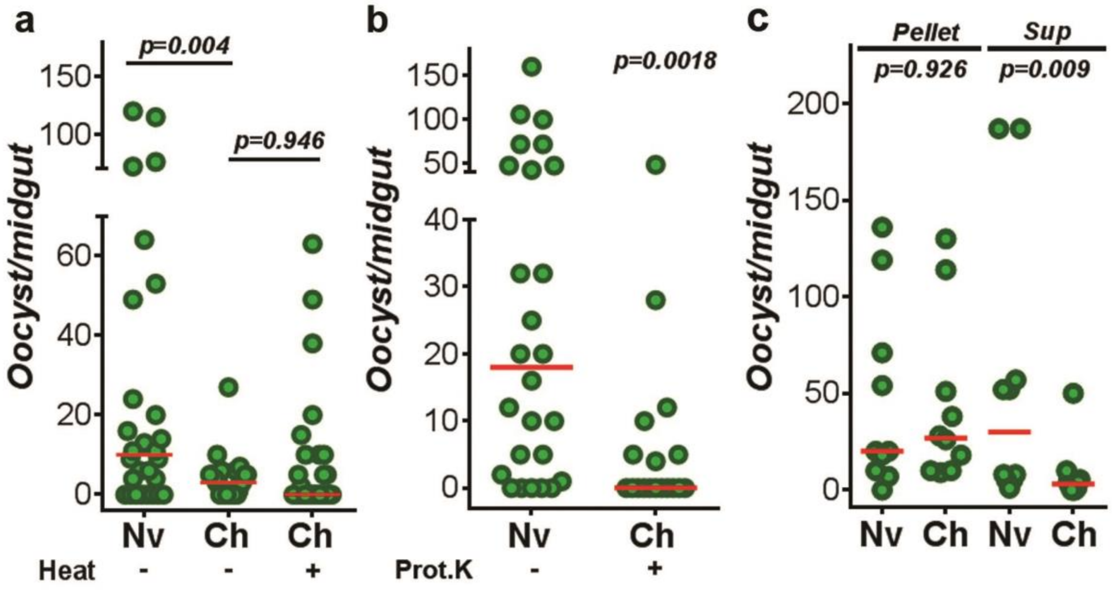

Supplementary Figure 1. **Biochemical characterization of HDF**. **(a)** Effect of Heattreatment and **(b)** Proteinase K (Prot.K) on HDF biological activity determined by its ability to enhance antiplasmodial immunity in recipient mosquitoes. **(c)** Effect of Heattreatment followed by ethanol precipitation on HDF biological activity determined by the ability to enhance antiplasmodial immunity in recipient mosquitoes. Nv: Naïve. Ch: Challenged. Sup: Supernatant. Nv: Naïve. Ch : Challenged. Each circle represents the number of parasites in an individual midgut and the red line indicates the medians. MannWhitney test, *, $p \leq 0.05$; **, $p \leq 0.01$; ***, $p \leq 0.001$; ****, $p \leq 0.0001$

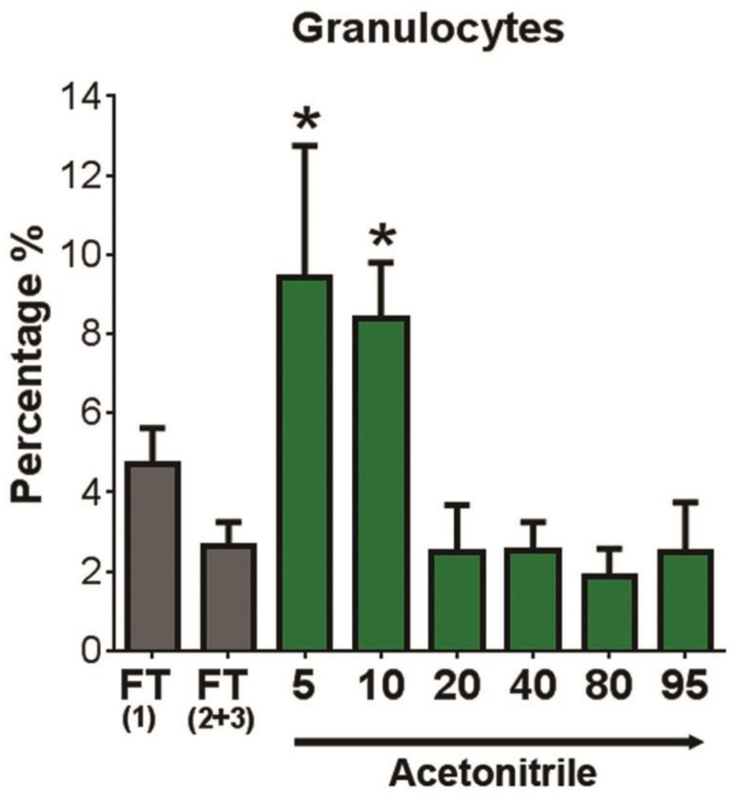

Supplementary Figure 2. HDF bioactivity in fractions after step-elution of a reversed-phase (C18) TARGA Minispin column with increasing concentrations of acetonitrile. HDF biological activity was determined by the ability to increase the percentage of granulocytes in recipient mosquitoes. FT1 = Flow-through and FT2+3 = the two subsequent washes with water prior to elution with acetonitrile. Error bars represent mean ± SEM. Granulocyte proportions were determined for each individual mosquito. Hemocytes were counted in 6 – 10 mosquitoes for each treatment and the results were confirmed in at least two independent experiments. Mann-Whitney test, *, $p \leq 0.05$.

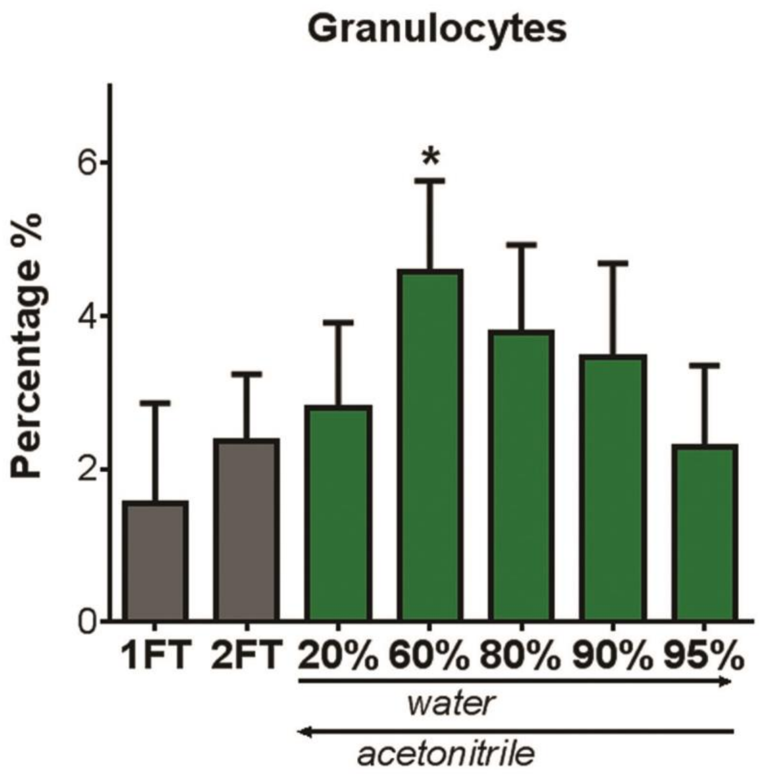

Supplementary Figure 3. HDF bioactivity in fractions after step-elution of a HILIC microtip column with increasing concentrations of water. HDF biological activity was determined by the ability to increase the percentage of granulocytes in recipient mosquitoes. 1FT= Flow-through and 2FT = subsequent wash with acetonitrile prior to elution with increasing concentrations of water. Error bars represent mean ± SEM. Granulocyte proportions were determined for each individual mosquito. Hemocytes were counted in 6 – 10 mosquitoes for each treatment and the results were confirmed in at least two independent experiments. Mann-Whitney test, *, $p \leq 0.05$.

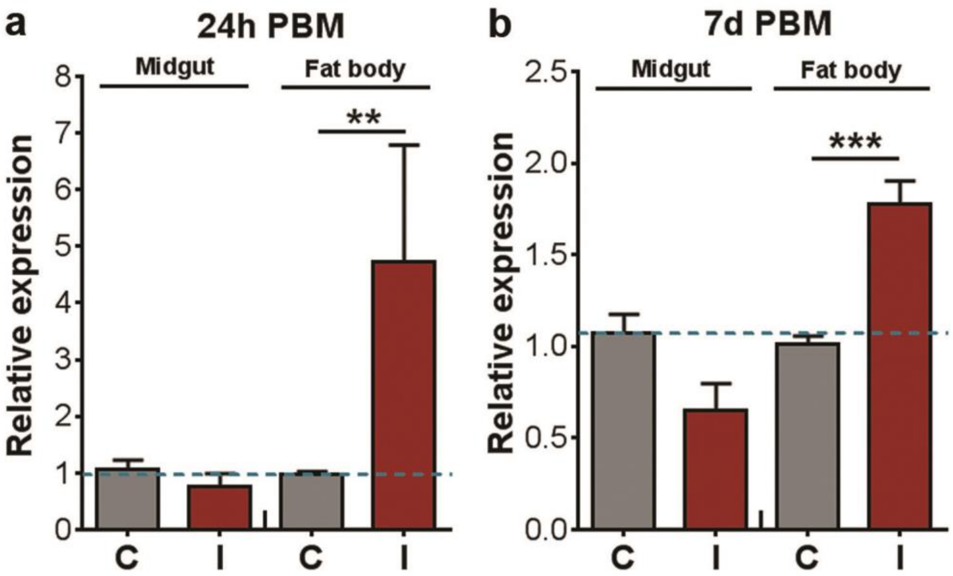

Supplementary Figure 4 . Evokin mRNA expression in the mosquito midgut and fat body following *P. berghei* infection. Evokin relative expression in the midgut and abdominal fat body at **(a)** 24 hours and **(b)** 7 days post-blood meal (PBM) on an control or *P. berghei*-infected mouse. Dotted blue line indicates basal levels of Evokin expression found in the control group. C: control; I: Infected. qRT-PCR mRNA expression analysis was done in two groups of midguts or body walls dissected from 15 mosquitoes (biological replicates) and analyzed in duplicate (technical replicates). The reproducibility of the result was confirmed in three independent experiments. Error bars represent mean ± SEM. Mann-Whitney test, **, $p \leq 0.01$; ***, $p \leq 0.001$.

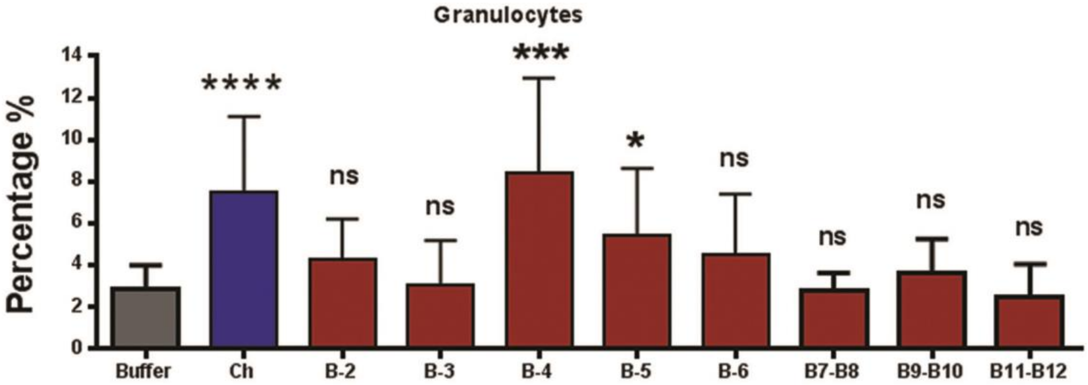

Supplementary Figure 5. HDF bioactivity of challenged hemolymph HILIC chromatography fractions. HDF biological activity was determined by the ability to increase the percentage of granulocytes in recipient mosquitoes, Activity in hemolymph from challenged mosquitoes (blue bar) and HILIC fractions (red bars). Injection of buffer alone was used as negative control (grey bar). Ch: Challenged. Error bars represent mean ± SEM. Granulocyte proportions were determined for each individual mosquito. Hemocytes were counted in 6 – 10 mosquitoes for each treatment and the results were confirmed in at least two independent experiments. Mann-Whitney test, *, $p \leq 0.05$; **, $p \leq 0.01$; ***, $p \leq 0.001$; ****, $p \leq 0.0001$; ns, $p>0.05$

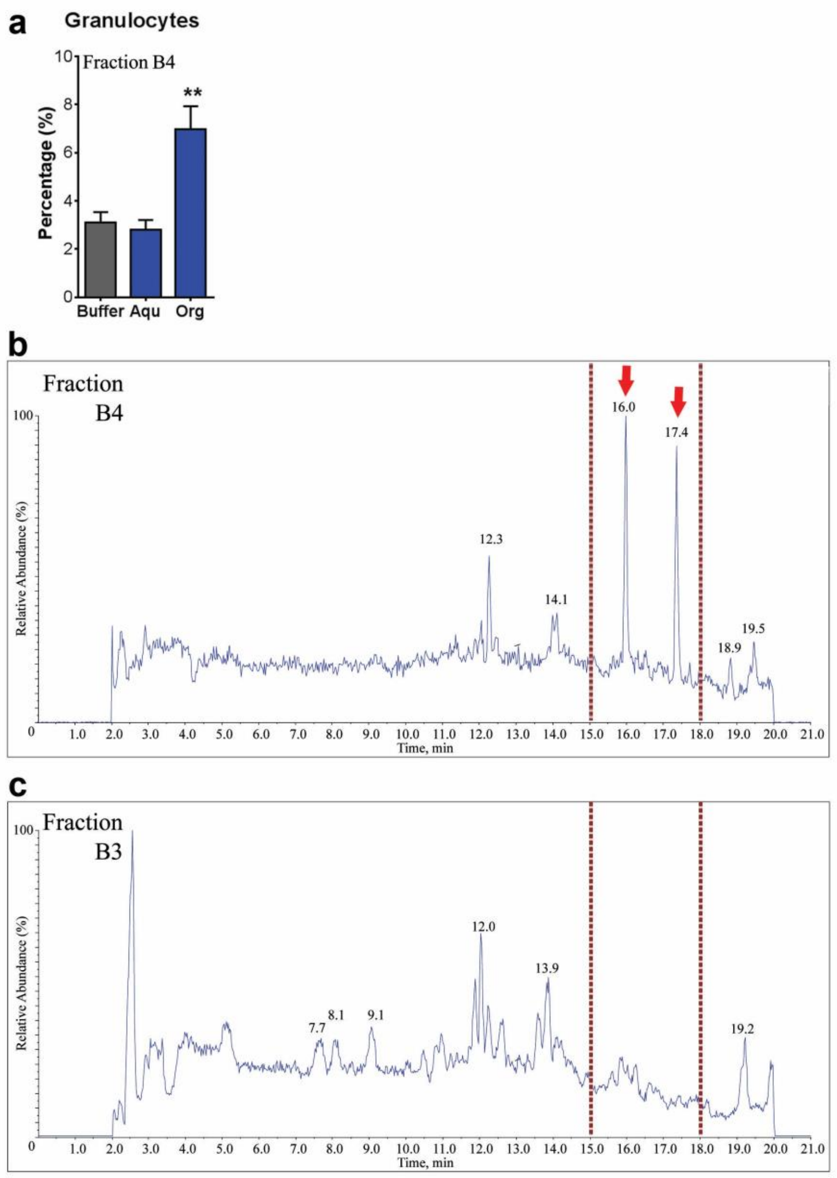

Supplementary Figure 6. HDF bioactivity and lipid mediator lipidomic (a) HDF bioactivity of HILIC fraction B4 following lipid extraction (Aqu: Aqueous phase; Org: Organic phase). Lipid mediator lipidomic chromatographs of **(b)** HILIC fraction B4 (highest HDF activity), and of **(c)** HILIC fraction B3 (lacking HDF activity). Red arrows indicate the two prominent peaks present in the fraction with HDF activity (organic phase of fraction B4). Error bars represent mean ± SEM. Granulocyte proportions were determined for each individual mosquito. Hemocytes were counted in 6 – 10 mosquitoes for each treatment and the results were confirmed in at least two independent experiments. Mann-Whitney test, **, $p \leq 0.01$

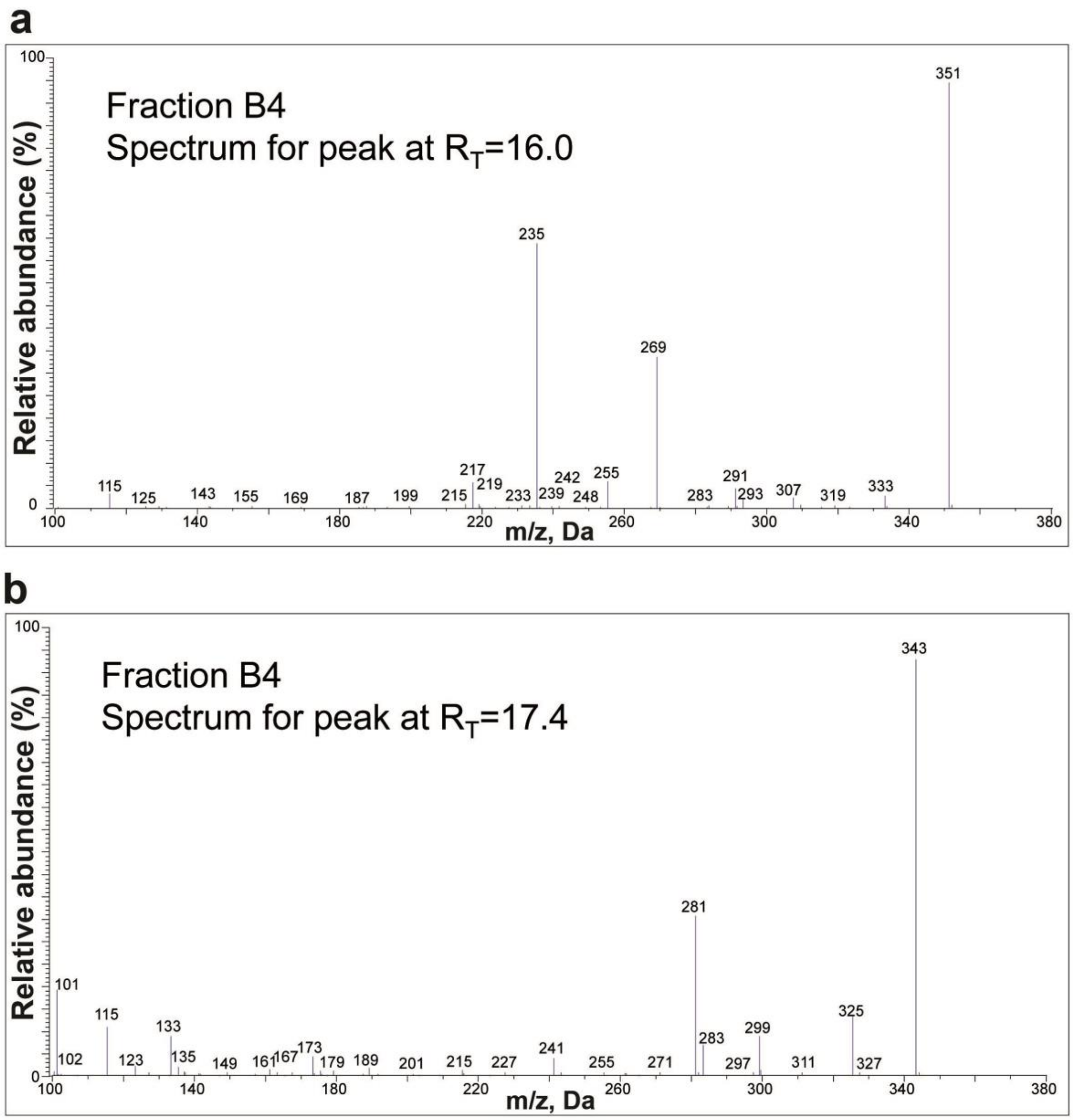

Supplementary Figure 7. LC MS/MS spectra of the two prominent peaks observed in fraction B4. (a) MS/MS spectrum of peak at Rt=16,0 ; a compound that shares mass spectrum signatures with vertebrate Lipoxins. **(b)** MS/MS spectrum of peak at Rt=17.4 ; a compound that shares mass spectrum signatures with 4-hydroxy-docosahexaenoic acid (4-HDHA).

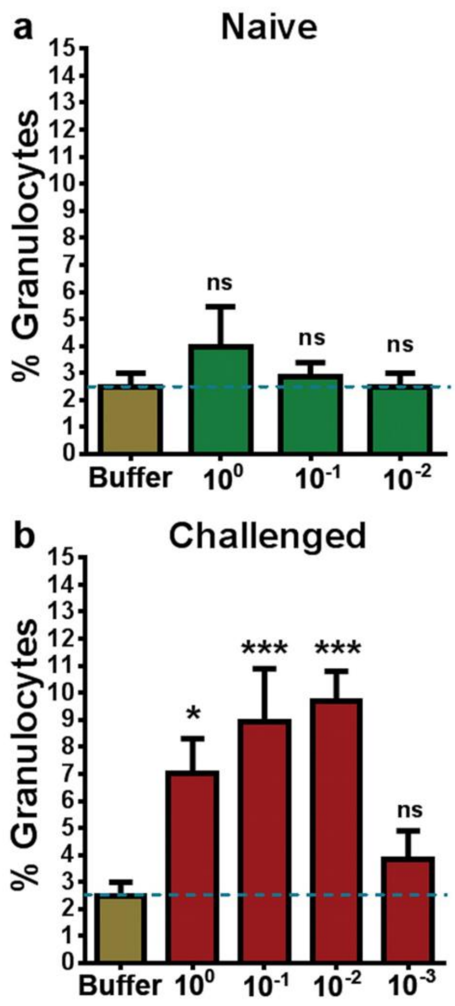

Supplementary Figure 8. Effect of systemic injection of deuterium-labeled arachidonic acid on HDF biological activity. Graphs represent the proportion of granulocytes in recipient mosquitoes in response to injection of different hemolymph concentrations (10-fold dilutions) from **(a)** naïve and **(b)** challenged donors. Donors were injected with d8-arachidonic acid 5 days post-feeding and hemolymph was collected 24h post-injection. Mosquitoes injected with buffer served as controls. Error bars represent mean ± SEM. Granulocyte proportions were determined for each individual mosquito. Hemocytes were counted in 6 – 10 mosquitoes for each treatment and the results were confirmed in at least two independent experiments. Mann-Whitney test, *, $p \leq 0.05$; **, $p \leq 0.01$; ***, $p \leq 0.001$; ns, $p>0.05$

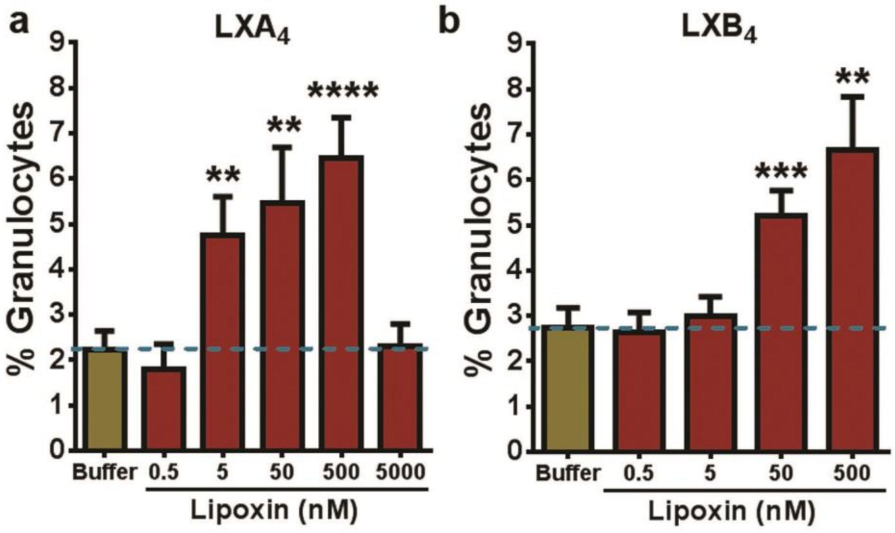

Supplementary Figure 9. Effect of systemic injection of synthetic Lipoxins on the proportion of granulocytes. Graphs represent the proportion of granulocytes in response to injection of different concentrations (10-fold dilutions) of **(a)** Lipoxin A_4 and **(b)** Lipoxin B_4. Mosquitoes injected with buffer served as controls and perfusions for hemocyte counting were done 4 days post-injection. Error bars represent mean ± SEM. Granulocyte proportions were determined for each individual mosquito. Hemocytes were counted in 6 – 10 mosquitoes for each treatment and the results were confirmed in at least two independent experiments. Mann-Whitney test, **, $p \leq 0.01$; ***, $p \leq 0.001$; ****, $p \leq 0.0001$.

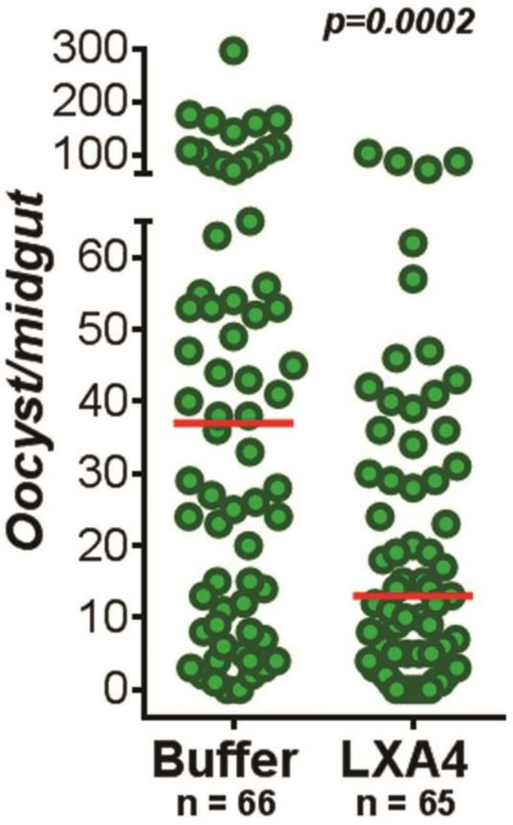

Supplementary Figure 10. Effect of synthetic Lipoxin A4 injection on *Plasmodium berghei* infection. Graph represents data from two independent feedings. Red horizontal line represents the median oocyst per midgut in buffer-injected (control) or LXA4injected mosquitoes. Each circle represents the number of parasites in an individual midgut and the line indicates the medians. Mann-Whitney test, ***; p=0.0002

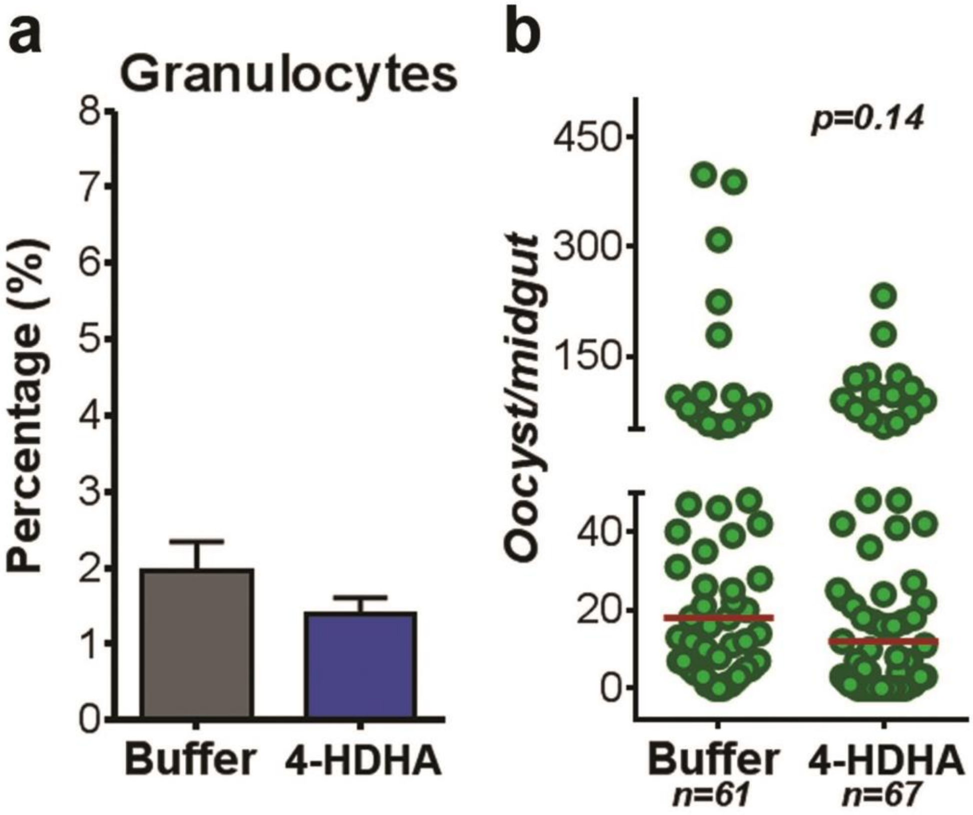

Supplementary Figure 11. Effect of synthetic 4-HDHA injection on (a) proportion of granulocytes and on **(b)** *Plasmodium berghei* infection. Graph represents data from two independent infections. Red horizontal line represents the median oocyst per midgut in buffer-injected (control) or 4-HDHA-injected mosquitoes. Error bars in fig. a represent mean ± SEM. Granulocyte proportions were determined for each individual mosquito. Hemocytes were counted in 6 – 10 mosquitoes for each treatment and the results were confirmed in at least two independent experiments. Each circle in figure b represents the number of parasites in an individual midgut and the line indicates the medians. MannWhitney test, ns, $p \geq 0.05$

Supplementary Table 1. Primer sequences used for Evokin silencing, gene expression analysis, and assessment of silencing efficiency.

Primer name	Primer sequence	AGAP -ID
**dsAG9281-F**	taatacgactcactatagggTGGCGGTACTCCTGCTACTC*	AGAP009281
**dsAG9281-R**	taatacgactcactatagggGCCGTTATACACTTCCCACC	AGAP009281

**Eff-2-9281-F**	CAGCCGAAAGTGAACAAACA	AGAP009281
**Eff-2-9281-R**	ATGGTGGCCATTGTATACGG	AGAP009281

*T7 promoter sequence is indicated in lower case letters.

Supplementary Methods

Quantitation of d_8-LXA_4 and d_8-LXB_4 in mosquito haemolymph

Arachidonic acid labelled with deuterium was injected to mosquitoes and its conversion into lipoxins was monitored. Briefly, 50 female mosquitoes from each experimental group (Naïve and Challenged) for Exp.1 and 80 per group for Exp. 2 were bled. Total lipid extraction from mosquito haemolymph was done after adding 400 pg of d5-Resolvin D2 to each sample, as internal reference to correct for the efficiency of the extraction. These samples were processed to quantitate d_8-LXA_4 and d_8-LXB_4 derived from deuterium-labelled arachidonic acid. Lipid extractions were carried out following the Bligh and Dyer method with slight modifications, and lipids were further separated according to their polarity using a pipette tip column of C18 resin, as indicated in the methods section. The methyl acetate and hexane fractions eluted from the C18 resin were dried under nitrogen gas, re-suspended in 100% methanol, stored at −80 °C and subjected to Lipid Mediator (LM)- lipidomics using liquid chromatography-tandem mass spectrometry (LC-MS-MS).

Step by Step Protocol on Deuterium-labelled Arachidonic Acid Injections and lipid extractions.

The following are step-by step protocol used to inject Deuterium-labeled arachidonic acid and the subsequent lipid extractions for MS/MS analysis.

Injection of Deuterium-labeled arachidonic acid.

1. Four-5-day-old mosquitoes were fed on either an infected or uninfected mouse, maintained at 19°C for 3 days prior their transfer to an incubator set at 27°C.
2. Mosquitoes were provided with oviposition cups at 3d post-blood meal.
3. At five days post-blood meal, mosquitoes were injected with 1µg of deuterated arachidonic acid (Arachidonic acid-d8) that was re-suspended in 1% BSA in Hanks Balanced Salt Solution (without Ca^{+2} and Mg^{+2}).
4. Mosquitoes were then cold-anesthetized and their hemolymph collected via perfusion from uninfected mosquitoes (Naïve) and infected mosquitoes (Challenged) at 24 h post-injection. Fifty female mosquitoes from each treatment group were perfused for Experiment 1 and 80 from each treatment for Experiment 2.

Spike with internal standard Resolvin D2-d_5

5. The collected hemolymph from Naïve and Challenged samples were spiked with 400 pg of Resolvin D2-d_5 as internal standard to calculate lipid extraction efficiency.

Lipid extraction via C18 fractionation

6. Lipid extraction on the spiked Naïve and Challenged samples were then conducted using the Blight and Dyer method (27) as stipulated in the methods section. Briefly, hemolymph samples were placed in a 5 ml glass tubes (Kimble Chase) and mixed with chloroform and methanol at a ratio 0.8:1:2 (V:V:V). The mixture was mixed by vortexing every 10 min for 1 h and then centrifuged at 580g for 10 min at room temperature. The supernatant was transferred to a new glass tube and mixed with an additional 0.5 ml of MIllii-Q water and 0.5 ml of chloroform and vigorously mixed in using a vortex. The mixture was centrifuged again at 580g for 10 min at room temperature to obtain a uniform phase separation. Both aqueous and organic phase were separately transferred to a 5 ml glass tube and taken to dryness with nitrogen gas. Dried samples were resuspended in 100% methanol for storage at -80 ^{o}C or further processing.
7. Lipid extracted samples were then further processed via reverse-phase (C18) separation using a pipette tip column of C18 resin (TARGA Reversed Phase, UltraMicroSpin, The Nest Group, Inc., Southborough, MA) as described in the methods section. Briefly, following conditioning of the C18 spin column, the column was loaded with either the aqueous or re-suspended organic phase, then spun or forced with a syringe. The column was washed once with HPLC-grade water prior to elution.
8. Elution of the C18 column was done by first eluting with 100ul of HPLC-grade methanol, followed by 100 ul of HPLC-grade methyl acetate and finally with 100 ul of HPLC-grade hexane.
9. The eluates from the methyl acetate and hexane fractions were dried under nitrogen gas then resuspended in 100% methanol and stored in amber glass vials at -80°C until used.
10. Samples were shipped overnight under dry ice to the Laboratory of Dr. Charles Serhan (Harvard University) for further lipid analysis via LC MS/MS.

LC MS/MS data analysis

Lipoxins deriving from chemically modified arachidonic acid were identified by MS/MS analysis based on six different diagnostic ions in lipids extracted from haemolymph of naïve and primed mosquitoes in two independent experiments. Because this was the first time that lipoxins were directly detected in insects, the presence of this specific combination of six diagnostic ions provided a molecular signature to ensure that lipoxins derived from the deuterium-labelled arachidonic acid substrate that had been provided to mosquitoes.

Only ions that corresponded to specific portions of the unlabelled lipid mediators, i.e. LXA_4 and LXB_4 were assigned. This approach is used to ensure that the assigned ions are derived from the mediator of interest rather than another molecule with a similar precursor ion. Given the low abundance of the biological material available, we did not have sufficient material to experimentally determine the origin of all the ions and assign each of them. Because the computer used to process data and prepare the original images

in figures 3e and 3f, is no longer available in 2020, the data were reprocessed from the original raw files obtained more than 5 years ago (see below). The relative abundance of the ions in the chromatograms shown may be slightly different from that in the published figures, because the region selected for background subtraction and for the spectrum itself may not be exactly the same. However, all key diagnostic ions identified in the MS/MS spectra displayed in the manuscript are also present in the raw spectrum provided below. The ions are presented as ion intensity and the key parameters used for identification of the different lipid mediators was the retention time and the presence of at least 6 diagnostic ions in the MS/MS spectrum.

Quantitation of lipoxins was performed by relating the area under the curve for a specific peak that corresponds to the mediator of interest to a standard curve obtained for this mediator or for a molecule with very similar physical properties. A LXA_4 standard curve was used to quantify both d_8-LXA_4 and LXB_4. Any losses during sample preparation were corrected by adding a specific internal standard that displays a similar chromatographic behaviour to the mediators of interest. The internal standard (d5-Resolvin D2) was added to each sample prior to processing and the amount present in the sample was determined using a similar procedure as outlined above for lipoxins. The percentage recovery of d5-Resolvin D2 internal standard in each of the sample was determined by dividing the amount of standard added by the amount of standard recovered and used to determine the absolute amount of mediators present in each sample.

Step by step protocol for Sample and Standard running in LC-MS/MS

1. Start the LC-MS/MS system
    a. An Agilent Poroshell 120 EC-C18 column (100 mm x 4.6 mm x 2.7 µm) is kept at 50°C
    b. The initial mobile phase consists of methanol/water/acetic acid 50:50:0.01 (vol/vol/vol)
    c. The flow rate is maintained at 0.5 mL/min
    d. The mobile phase is ramped to 80:20:0.01 (vol/vol/vol) from 2 min to 11 min
    e. The mobile phase is maintained till 14.5 min
    f. The mobile phase is ramped to 98:2:0.01 (vol/vol/vol) for the next 0.1 min
    g. The mobile phase is maintained at 98:2:0.01 (vol/vol/vol) for 5.4 min

2. A method employing the following Multiple Reaction Monitoring (MRM) ions was employed for the identification and quantitation of d_5-RvD2, LXA_4 and LXB_4
    a. For d_5-RvD2 - 380.2 > 141.0
    b. For d_8-LXA_4 – 359 > 116.1; 359 > 224.1;
    c. For d_8-LXB_4 – 359 > 227.1; 359 > 341.1
3. Enhanced Product Ion scans were coupled with the MRM experiment using Information Dependent Acquisition experiment with for ions between 100-400 m/z.
4. A mix of synthetic standards containing d_5-RvD2, LXA_4 and LXB_4 was injected to establish the retention of the deuterium labelled internal standard (d_5-RvD2) and the molecules of interest (LXA_4 and LXB_4).

5. The samples were then injected
6. The 100% standard, which is used to calculate extraction recoveries to facilitate the quantitation of mediators, was then injected

Step-by-step protocol for chromatographic identification of mediators and figure preparation

1. Data was acquired using Analyst software (version 1.6, Framingham, MA) and a QTRAP 5500 Sciex equipped with LC-20AD HPLC Shimadzu.
2. Laboratory standards were run on LC-MS/MS before each set of samples to determine their retention time. These retention times are also determined in relation to the appropriate deuterium internal standard
3. Targeted analytes are flagged by matching the retention time with corresponding authentic standard.
4. The multiple reaction monitoring (MRM) trace is then saved as an active text file by right clicking on the screen
5. The text file, with the retention times (x axis/left column) and the peak area (y axis/right column), is opened and the data copied and pasted into a GraphPad Prism software XY table.
6. The text file, with the retention times (x axis/left column) and the peak area (y axis/right column), is opened and the data copied and pasted into a GraphPad Prism software XY table.
7. The corresponding XY graph is then edited to remove the ticks on the Y axis and to format the X axis from 0 to 22 (minutes).
8. The XY graph generated in GraphPad Prism is then copied and passed into PowerPoint Software
9. The chromatographic trace is ungrouped
10. This is then placed on to a template 3D frame

Step-by-step protocol for MS/MS spectra matching and figure preparation

1. Data was acquired using Analyst software (version 1.6, Framingham, MA) and a QTRAP 5500 Sciex equipped with LC-20AD HPLC Shimadzu.
2. From the total ion chromatogram (TIC), the enhanced product ion (EPI) spectra is identified in the chromatographic region corresponding with the retention time of the mediator of interest.
3. A region immediately before or after the region of interest is then set for background subtraction
4. The MS/MS spectrum is then centroided.
5. The spectrum is then copied and transferred into the PowerPoint software
6. The spectrum is then ungrouped to highlight the diagnostic ions of corresponding with those assigned for the mediator of interest in a reference standard.
7. Ion labels that are not assigned are removed.
